# Computational Modeling of Stereotype Content in Text

**DOI:** 10.3389/frai.2022.826207

**Published:** 2022-04-19

**Authors:** Kathleen C. Fraser, Svetlana Kiritchenko, Isar Nejadgholi

**Affiliations:** National Research Council Canada, Ottawa, ON, Canada

**Keywords:** stereotypes, natural language processing, computational social science, computational model, sentence embeddings, social media analysis, text analysis, biased language

## Abstract

Stereotypes are encountered every day, in interpersonal communication as well as in entertainment, news stories, and on social media. In this study, we present a computational method to mine large, naturally occurring datasets of text for sentences that express perceptions of a social group of interest, and then map these sentences to the two-dimensional plane of perceived *warmth* and *competence* for comparison and interpretation. This framework is grounded in established social psychological theory, and validated against both expert annotation and crowd-sourced stereotype data. Additionally, we present two case studies of how the model might be used to answer questions using data “in-the-wild,” by collecting Twitter data about women and older adults. Using the data about women, we are able to observe how sub-categories of women (e.g., Black women and white women) are described similarly and differently from each other, and from the superordinate group of women in general. Using the data about older adults, we show evidence that the terms people use to label a group (e.g., old people vs. senior citizens) are associated with different stereotype content. We propose that this model can be used by other researchers to explore questions of how stereotypes are expressed in various large text corpora.

## 1. Introduction

Stereotypes are pervasive in our society. The term *stereotype* refers to the cognitive representation people hold about a social group, consisting of beliefs and expectations about probable traits and behaviors (Beukeboom and Burgers, 2019). By categorizing people into groups, and then making assumptions about individuals on the basis of their group membership, we are able to make predictions about the world. However, stereotypes can be dangerous when they prevent individuals from being seen for who they are, rather than according to the over-simplified perceptions of the group as a whole.

Language plays an important role in the communication of stereotypes. The linguistic *content* of statements about certain social groups is one source of information, and various theories of social cognition seek to describe and explain stereotype content. One such theory is the Stereotype Content Model (SCM) (Fiske et al., 2002, 2006), wherein stereotypes are decomposed into the two primary dimensions of *warmth* (whether a group is perceived as being social, moral, and cooperative) and *competence* (whether a group is perceived as being capable and agentic). The SCM thus proposes that many groups are not stereotyped as simply “good” or “bad,” but can be simultaneously ranked highly on one dimension and low on the other, resulting in complex social relationships. For example, Asian Americans are often stereotyped as highly competent and academically successful, but lacking warmth and sociability, leading to envious prejudice. This is in contrast to, for example, people with drug addictions, who are seen as both antisocial and incapable of productive action, and who are therefore viewed with disgust rather than envy. Numerous survey-based studies have provided evidence for the hypotheses of the SCM, across multiple cultures and social gro ups. However, even beyond stereotype content, other linguistic cues can convey stereotypic information, including the *labels* used to categorize and sub-divide different social groups (Beukeboom and Burgers, 2019).

While psychological researchers have studied stereotypes for decades, computer scientists in the field of natural language processing (NLP) have only started exploring this area. As we will discuss, much of the NLP work has focused on detecting and mitigating stereotypical bias in NLP tools, such as word embeddings and large-scale language models. Here, we focus instead on detecting human biases, using computational techniques to analyze social media data within the established framework of the SCM. In contrast to preliminary work which operated only on the word level, and was thus restricted to manually-generated data (Fraser et al., 2021), we here extend our computational model to the sentence level. We build synthetic training sentences labeled for combinations of warmth and competence from an annotated lexicon. Then, we optimize and refine the trained model to achieve high accuracy in mapping various semantic and syntactic forms of stereotypical sentences to the two-dimensional SCM plane. We then validate our model in two tasks: (1) reproducing continuous scale SCM scores generated by manual annotations, and (2) reproducing group level stereotypes reported in the literature, given crowd-sourced stereotypical sentences about those groups. The model and associated data are publicly available for the use of other researchers^1^.

Finally, based on our computational model, we introduce a general framework for uncovering stereotypical views about a group of interest in a particular data source. We demonstrate, with two case studies, how our computational model could be used to study widespread perceptions of social groups on Twitter, focusing on how women and older adults are portrayed on social media. We analyze the results of the case studies with reference to known aspects of stereotyping, such as subtyping and category labeling.

## 2. Background and Related Work

We begin with a discussion of the SCM and related theories from the social psychology literature, followed by a summary of the related work in the areas of natural language processing and machine learning. We then situate the current work within these intersecting areas of research and describe the goals of the present study.

### 2.1. Psychological Models of Stereotype Content

In contrast to early stereotype research, which focused on negative stereotypes and studied them in a binary *us vs. them* framework (Allport et al., 1954), more recent models of social cognition, such as SCM (Fiske et al., 2002), Agency-Beliefs-Communion Model (Koch et al., 2016), Dimensional Compensation Model (Yzerbyt, 2018), Dual Perspective Model (Abele and Wojciszke, 2007), and Behavioral Regulation Model (Leach et al., 2007), involve several dimensions, creating room for ambivalent out-group orientations. In this work, we focus on SCM, while emphasizing that our computational methodology can be trivially extended to higher or differently-defined dimensions.

The SCM proposes the two principal dimensions of *warmth* and *competence* to represent stereotypes. According to the SCM, evolution predisposed us to form a quick cognitive representation of strangers by first assessing whether they intend to harm us, captured in the primary dimension of warmth, and then judging if they are capable of acting on the perceived intention, reflected on the competence dimension. An important aspect of the SCM is its ambivalent stereotypes hypotheses; that is, that many groups are stereotyped as being high on one dimension and low on the other. For example, in American society, rich and powerful businesspeople may be stereotyped as competent but cold, while grandparents or homemakers are stereotyped as warm but not competent (Fiske, 2018). Of course, some groups are also stereotyped as high on both dimensions (e.g., the middle class) or low on both dimensions (e.g., homeless people). The four quadrants defined by the SCM not only *describe* stereotypes, but can be linked to a causal framework in which elements of social structure such as status and interdependence *predict* perceived competence and warmth, respectively (Fiske, 2015). Moreover, stereotypes then predict emotional prejudices, with groups perceived as high-competence, high-warmth eliciting admiration, groups perceived as warm but incompetent eliciting pity, groups perceived as competent but cold eliciting envy, and those in the low-low quadrant eliciting disgust. The Behavior from Intergroup Affect and Stereotypes (BIAS) Map then extends the SCM to link emotions with actions and behaviors (Cuddy et al., 2007).

The SCM has been applied and tested in many different scenarios. In a cross-cultural study involving three East Asian and seven European countries, Cuddy et al. (2009) showed that the SCM hypotheses applied almost universally. The SCM has also been used to study stereotypes relating to gender (Eckes, 2002; Cuddy et al., 2008; Johnson et al., 2018), race (Lin et al., 2005; Grigoryev et al., 2019), immigration status (Lee and Fiske, 2006), and social class (Durante et al., 2017). Recent work has also proposed novel applications, such as quantifying human impressions of artificial intelligence agents (McKee et al., 2021).

In previous work, we proposed a word-level computational model of the SCM (Fraser et al., 2021). This work leveraged the lexicons made available by Nicolas et al. (2021), for which they manually labeled several hundred words as being associated with theoretically-motivated dimensions of stereotype content, including agency, ability, sociability, and morality. They then used this manually-labeled “seed lexicon” to generate a large, automatically-labeled “extended lexicon” of words associated with warmth and competence, as well as various other psychological constructs. We adapted the POLAR framework (Mathew et al., 2020), which uses semantic differentials to interpret word embeddings on a scale between two polar opposites, to develop a tool to project words onto the two-dimensional warmth-competence plane. We demonstrated that our word-level model, trained on Nicolas et al. (2021)'s lexicon, was able to associate words from the extended lexicon with the expected polarities of warmth or competence.

### 2.2. NLP Techniques for Stereotype Detection

Many research studies in NLP have focused on the stereotypical biases encoded in word embeddings trained on large text corpora, for example showing that the word vector for *woman* and *homemaker* are close, while the vector for *man* is close to that of *computer programmer* (Bolukbasi et al., 2016; Caliskan et al., 2017). More recently, work has focused on detecting stereotypical associations present in large-scale language models (Abid et al., 2021; de Vassimon Manela et al., 2021). As a tool for evaluating such stereotypical bias, datasets of common stereotypes have been manually created, including StereoSet (Nadeem et al., 2020) and CrowsPairs (Nangia et al., 2020). Other work in NLP has aimed to mitigate stereotypical biases in language technologies (Sun et al., 2019; Zmigrod et al., 2019). However, identifying *human* stereotypes from text is a relatively under-explored area.

Among unsupervised NLP techniques, lexicon-based sentiment analysis and statistical measures of word co-occurrence have been used to address some aspects of this topic. Rudinger et al. (2017) investigated stereotypical biases in elicited text using pointwise mutual information and qualitative examples, finding gendered associations between the prompts and texts. Marzouki et al. (2020) identified shifting stereotypes of Muslim people in the aftermath of the Charlie Hebdo attacks, by measuring the positive and negative valence of words frequently co-occurring with a set of key terms such as *Islam, Muslim*, and *Prophet*. With the emergence of word embeddings as models encoding the semantics of language, embedding-based unsupervised techniques have also been developed to explore biased language (Garg et al., 2018; Charlesworth et al., 2021).

Supervised learning of stereotypes has been also explored in NLP, often in the context of detecting abusive behavior. While high levels of performance have been achieved in identifying abusive content containing explicitly obscene expressions, identifying more subtly expressed abuse, such as stereotyping and micro-aggression, has proven to be challenging (Breitfeller et al., 2019; Caselli et al., 2020). Toward this goal, Fersini et al. (2018) and Chiril et al. (2020) examined gender-related stereotypes as a sub-category of sexist language, and Price et al. (2020) annotated “unfair generalizations” as one attribute of unhealthy online conversations. Cryan et al. (2020) used supervised classifiers as well as lexicon-based techniques to detect gender stereotypes in text. Sap et al. (2020) annotated a large corpus of abusive online posts for the implied stereotypical meaning and showed that the current generative models struggle to effectively reproduce human interpretations of the stereotypical views expressed in implicit abuse. The current state of the field is summarized by Wiegand et al. (2021), who identified stereotypes as one of the sub-types of implicitly abusive language that is not learned well by current abusive language detection models and that requires new datasets with a revised task formulation, data sampling strategies, and annotation schemes.

Other NLP studies have adopted insights from the social sciences to explore how stereotyping is reflected in language. For example, Joseph et al. (2017) clustered tweets about racially-motivated police brutality according to two theories of stereotyping, Affect Control Theory and Semantic Relationship Theory, to explain stereotypes across two dimensions: evaluation (good/bad) and potency (strength/weakness). In another study, Fokkens et al. (2018) extracted *micro-portraits*—impressions of a target group or an individual conveyed in a single text—to explore stereotypes about Muslim men in Dutch media. Lee et al. (2019) presented two chatbots with stereotypical statements from psychological surveys, and assessed whether the chatbots agreed or disagreed with the statements using a textual entailment model.

In contrast to these works, our goal is to develop a general computational framework that combines the information encoded in embedding models with the SCM theory, and allows us to analyse and compare various stereotypes in the shared space of warmth and competence. This tool can be used for mining naturally occurring text data, and does not require annotations or pre-existing assumptions about the stereotypic views that may be expressed in the text. Also, we make use of pretrained embedding models; in contrast to previous studies that *train* embedding models to uncover stereotypes, our method is not limited to analysis of extremely large corpora and is not demanding in terms of computational power. The model builds upon our preliminary work as described by Fraser et al. (2021), which operated only at the word level. We consider the extension to sentence level to be an essential step toward analyzing stereotypes in natural language datasets, since most text data exists in the form of sentences, and extracting only the relevant words for analysis is nontrivial. The sentence-level model is able to take into account important information from the textual context, including negation markers, grammatical conjunctions, and so on. In the following sections, we present an extensive validation of the model, and then demonstrate its potential use in two case studies, examining gender- and age-based stereotypes.

## 3. Materials and Methods

In the following, we describe our computational model in stages, from the mathematical framework underpinning the model, to the development of the model and the integration of various refinements and improvements, and finally to testing and model selection based on a test suite of desired linguistic capabilities. From there, we validate the selected model against human annotations of warmth and competence. We then use the model to compute warmth and competence values for sentences from a corpus of stereotypes, aggregate those values to acquire overall warmth and competence scores for different social groups, and then compare those values with findings reported in survey-based psychological studies.

### 3.1. Model Development

#### 3.1.1. Word-Level Model

We aim to extend the word-level model, described in Fraser et al. (2021), to the sentence level. We describe the details of the word-level model here. To define each of the directions warmth, coldness, competence, and incompetence, we consider the set of adjectives associated with each direction in the seed lexicon provided by Nicolas et al. (2021). Specifically, we include all adjectives from the *sociability* and *morality* dictionaries to define positive and negative warmth, and all words from the *agency* and *ability* dictionaries to define positive and negative competence. Our approach is in contrast to the standard POLAR framework introduced by Mathew et al. (2020), which considers word *pairs*, rather than *sets*. Therefore, we use a slightly different formulation to obtain the polar directions associated with warmth and competence^2^.

Let $D=\left[\vec{W_{1}^{a}},\vec{W_{2}^{a}},\vec{W_{3}^{a}},...,\vec{W_{V}^{a}}\right]\in {\mathbb{R}}^{V\times d}$ denote the set of pretrained *d*-dimensional word embedding vectors, trained with algorithm *a*, where *V* is the size of the vocabulary and $\vec{W_{i}^{a}}$ is a unit vector representing the *i*^*th*^ word in the vocabulary.

In the word-level model, we use four sets of seed words; a set of *N*_1_ words associated with positive warmth ${\mathbb{P}}_{w+}=\left\{p_{w+}^{1},p_{w+}^{2},...,p_{w+}^{N_{1}}\right\}$, a set of *N*_2_ words associated with negative warmth, ${\mathbb{P}}_{w-}=\left\{p_{w-}^{1},p_{w-}^{2},...,p_{w-}^{N_{2}}\right\}$, a set of *N*_3_ words associated with positive competence, ${\mathbb{P}}_{c+}=\left\{p_{c+}^{1},p_{c+}^{2},...,p_{c+}^{N_{3}}\right\}$, and a set of *N*_4_ words associated with negative competence, ${\mathbb{P}}_{c-}=\left\{p_{c-}^{1},p_{c-}^{2},...,p_{c-}^{N_{4}}\right\}$. In order to find the two polar opposites, we obtain the following directions:


(1) $$\begin{array}{l} \vec{dir_{1}}=\frac{1}{N_{1}}\sum_{i=1}^{N_{1}}W_{p_{w+}^{i}}^{a}-\frac{1}{N_{2}}\sum_{i=1}^{N_{2}}W_{p_{w-}^{i}}^{a}\\ \vec{dir_{2}}=\frac{1}{N_{3}}\sum_{i=1}^{N_{3}}W_{p_{c+}^{i}}^{a}-\frac{1}{N_{4}}\sum_{i=1}^{N_{4}}W_{p_{c-}^{i}}^{a} \end{array}$$

where $W_{\upsilon }^{a}$ represents the vector of the word υ. The two direction vectors are stacked to form *dir* ∈ ℝ^2 × *d*^, which represents the change of basis matrix for the new two-dimensional embedding subspace E. In the new subspace, a word υ is represented by ${\vec{E}}_{\upsilon }$, which is calculated using the following linear transformation:


(2) $${\vec{E}}_{\upsilon }={\left(dir^{T}\right)}^{-1}W_{\upsilon }^{a}$$

Each dimension in E can now be interpreted in terms of the polar opposites used to define $\vec{dir_{1}}$ and $\vec{dir_{2}}$; in this case, warmth-coldness and competence-incompetence.

#### 3.1.2. Sentence-Level Model

To extend the model to the sentence level, we use sentence embeddings in the place of word embeddings. We first replace the sets of training *words* with sets of training *sentences* by inserting each seed word into a sentence template, such as: *These people are always [BLANK]*, where *[BLANK]* can be filled with any of the adjectives from the seed lexicon. As an example, if the word *warm* was a seed word in the set ℙ_*w*+_ above, then the sentence-level model would instead include *These people are always warm* in its training set ℙ_*w*+_. The text sentences are transformed into embeddings using a pretrained sentence embedding model, as described below in Section 3.1.3. Then the method proceeds as before, with $W_{\upsilon }^{a}$ now representing the vector of the sentence υ.

Although the basic sentence-level model as described above works reasonably well, we wanted to investigate whether the model could be improved for our specific, two-dimensional case. We considered two possible methods of improving on the base model: choosing a different set of basis vectors for the vector transformation (Equation 2), and reducing the dimensionality of the sentence embeddings before projecting them down to the warmth-competence plane. We motivate and explain these modifications below.

As Mathew et al. (2020) demonstrate, the POLAR framework performs better in low dimensions when the polar opposite vectors are maximally orthogonal. Here, we consider only two dimensions (warm–cold and competent–incompetent), leading to the following problem with respect to orthogonality: In the seed lexicon, words are annotated for only warmth *or* competence, meaning the opposite dimension is ill-defined. Thus, while we might naively assume that all the sentences containing high-competence seed words should be mapped to (1,0), and all the sentences containing high-warmth words to (0,1), we do not actually know that this to be the case. In fact, we observe a negative correlation between sentence vectors representing warmth and competence (see the Supplementary Materials for an visualization of this phenomenon).

However, working on the sentence level (rather than the word level) suggests a solution to this problem: we can define basis sentences that contain *two* words from the seed lexicon, one with a known competence value and one with a known warmth value. For example, *These people are always smart and friendly* should be mapped to (1,1), and *These people are always stupid and cruel* should be mapped to (−1, −1). Therefore, we can alternatively use these sentences with *two* seed words to increase the orthogonality of the training pairs and potentially improve performance of the model. For ease of interpretation, as a final step in the algorithm we then simply rotate the projected data by 45° so that they align with the usual axes representing high competence as (1, 0) and high warmth as (0, 1). For this reason, we call this modification “axis rotation.”

The second modification that we consider is an intermediate dimensionality reduction step. High-dimensional sentence embeddings contain much information which is irrelevant to the determination of warmth and competence. To uncover the most relevant latent dimensions, we consider two standard methods of dimensionality reduction: principal components analysis (PCA) (Wold et al., 1987; Gewers et al., 2021), which takes an unsupervised approach to determine the dimensions which explain the highest variance in the data, and partial least squares (PLS), which performs a similar function but in a supervised fashion (Garthwaite, 1994; Rosipal and Krämer, 2005). In each case, we fit the dimensionality reduction model on the same sentences that occur in the POLAR training data. The number of dimensions is set to 10.

Finally, there are a wide variety of sentence embedding models which can be used to encode the text sentences as vectors. We consider here a set of pretrained models available on the HuggingFace Sentence Transformer page^3^. Specifically, we experiment with RoBERTa sentence embeddings (Liu et al., 2019) pretrained for three general NLP tasks: semantic textual similarity (STS), natural language inference (NLI), and paraphrase mining. As baseline models, we also consider averaged GloVe word embeddings (Pennington et al., 2014) as well as the MPNet sentence embedding model (Song et al., 2020) recommended as the best “general purpose” sentence embeddings^4^.

#### 3.1.3. Model Selection

In the previous section, we described a variety of design decisions which affect the final performance of the model: axis-rotation, dimensionality reduction, and sentence embedding model. Here, we aim to determine the optimal combination of these variables such that our model can accurately predict warmth and competence in a variety of linguistic constructions. This will allow us to select the best model to use in the rest of our experiments. We conduct four evaluations of increasing complexity, to determine the linguistic capabilities of each embedding model (as summarized in Table 1):

- **Basic functionality:** The ability of the system to correctly predict the polarity (high/low) of a dimension for each sentence generated from the template *These people are always [BLANK]*, where *[BLANK]* is replaced with an adjective from the seed lexicon. The gold label for the sentence corresponds to the label of the adjective in the seed lexicon.
- **Negation:** The ability of the system to correctly predict the polarity (high/low) of a dimension for each *negated* sentence generated from template *These people are never [BLANK]*, where *[BLANK]* is replaced with an adjective from the seed lexicon. The gold label for the sentence corresponds to the opposite of the adjective's label in the seed lexicon. Negation is a common linguistic phenomenon that can be challenging for some automatic methods, e.g., the ones based on lexicon matching.
- **Semantic composition:** The ability of the system to correctly predict the polarity (high/low) of both dimensions (i.e., the correct quadrant) for each sentence generated from template *These people are always [BLANK] and [BLANK]*, where *[BLANK]*s are replaced with two adjectives from the seed lexicon, one with a warmth label and one with a competence label. The gold labels for the sentence corresponds to the labels of the adjectives in the seed lexicon.
- **Syntactic variability:** The ability of the system to correctly predict the polarity (high/low) of both dimensions (i.e., the correct quadrant) for each sentence generated from varying templates of the form *[Subject phrase] [BLANK] [connector] [BLANK]*, in which *[BLANK]*s are replaced with two adjectives from the seed lexicon, one with a warmth label and one with a competence label. The *subject phrase* and *connector* are randomly chosen from a set of five and seven options, respectively, leading to syntactically complex sentences such as the example in Table 1. The gold labels for the sentence corresponds to the labels of the adjectives in the seed lexicon.

**Table 1:** Testing the linguistic capabilities of each model.

**Capability**	**Metric**	**Sample test case**
Basic functionality	1D accuracy	These people are always friendly (Label: warm)
Negation	1D accuracy	These people are never friendly (Label: cold)
Semantic composition	2D accuracy	These people are always friendly and smart (Label: warm and competent)
Syntactic variability	2D accuracy	This group is known for being friendly as well as smart (Label: warm and competent)

*While the models always predict two values (warmth and competence) for each sentence, the lexicon data provide gold labels for only one dimension (warmth or competence). Therefore, in the first two cases, each test case has only one gold label, and so accuracy is measured by whether the model correctly assigns positive vs. negative warmth or competence. In the last two cases, each test case is associated with gold values for both warmth and competence dimensions, and so the accuracy is measured for both dimensions (i.e., the model must place the sentence in the correct quadrant)*.

We evaluate the models using five-fold cross-validation, where in each case we use 80% of the seed words to generate the training sentences, and use the remaining 20% of the words to generate the test sentences. Note that the training sentences always take the same format; only the test sentences change in the four functional evaluations. For standard POLAR the training sentences take the form, *These people are always [BLANK]*, while for axis-rotated POLAR they take the form, *These people are always [BLANK] and [BLANK]*. The complete labeled test data are available in the Supplementary Material.

The results of the cross-validation experiments are given in Table 2. The RoBERTa models trained on STS and NLI datasets perform the best, with the NLI model generally performing the best overall. The GloVe baseline performs remarkably well on the basic functionality, but fails to properly handle negation and syntactic variation. Across the four functional test cases, the axis-rotated POLAR model with PLS dimensionality reduction leads to the highest accuracy in three out of four cases, with the fourth case (negation) being handled best by the axis-rotated model with PCA. Therefore, in all the work that follows we use the RoBERTa model trained on NLI data (*roberta-nli*), with axis-rotated POLAR and PLS dimensionality reduction.

**Table 2:** Mean accuracy (with standard deviation in parentheses) across folds for each combination of model, configuration, and functional test category.

**Function**	**Model**	**Standard POLAR**			**Axis-rotated POLAR**		
		**None**	**PCA**	**PLS**	**None**	**PCA**	**PLS**
Basic	RoBERTa-STS	94.5 (2.1)	93.6 (1.8)	95.4 (2.0)	95.3 (2.0)	95.3 (2.0)	96.2 (0.8)
	RoBERTa-NLI	**95.0 (3.8)**	**95.4 (3.3)**	**96.2 (2.8)**	***97.9 (2.3)***	***97.9 (2.3)***	***97.9 (2.3)***
	RoBERTa-para	92.7 (3.6)	90.2 (3.2)	92.3 (3.5)	95.3 (0.8)	95.3 (1.7)	94.5 (1.5)
	GloVe-average	90.2 (5.5)	80.1 (5.6)	90.2 (4.3)	92.2 (3.2)	91.4 (3.7)	92.3 (2.9)
	MPNet-para	92.3 (2.4)	94.0 (3.6)	95.3 (2.5)	95.4 (2.0)	94.0 (2.2)	95.7 (0.2)
Negation	RoBERTa-STS	91.4 (3.8)	92.3 (2.4)	94.0 (2.5)	93.1 (2.9)	92.3 (3.2)	93.2 (3.2)
	RoBERTa-NLI	**95.3 (1.6)**	**95.3 (2.8)**	**95.3 (1.6)**	**95.8 (3.1)**	***96.2 (2.4)***	**95.8 (2.6)**
	RoBERTa-para	91.1 (2.3)	88.1 (2.3)	91.5 (1.2)	91.6 (4.5)	92.4 (2.3)	92.8 (2.0)
	GloVe-average	9.8 (5.5)	19.9 (5.6)	9.8 (4.3)	7.8 (3.2)	8.6 (3.7)	7.7 (2.9)
	MPNet-para	94.0 (5.5)	92.7 (6.9)	91.2 (4.7)	94.5 (3.9)	94.9 (3.9)	93.3 (3.2)
Semantic	RoBERTa-STS	**73.9 (9.8)**	76.8 (10.0)	75.0 (8.8)	76.8 (10.1)	76.9 (12.0)	78.7 (7.6)
	RoBERTa-NLI	**73.9 (9.8)**	**77.9 (8.2)**	**77.7 (10.3)**	**81.6 (8.2)**	**78.8 (7.9)**	***84.4 (7.7)***
	RoBERTa-para	64.4 (7.2)	61.4 (5.2)	67.3 (9.3)	57.8 (7.2)	58.6 (4.4)	57.8 (8.7)
	GloVe-average	62.5 (7.9)	51.1 (8.1)	71.0 (7.5)	67.3 (8.5)	63.5 (8.2)	65.3 (7.0)
	MPNet-para	58.5 (11.5)	62.4 (8.5)	67.4 (10.7)	57.9 (7.0)	61.6 (2.0)	62.6 (5.6)
Syntactic	RoBERTa-STS	69.2 (10.3)	**70.1 (14.7)**	**73.2 (14.0)**	**73.1 (12.1)**	**72.2 (10.8)**	75.1 (13.1)
	RoBERTa-NLI	**70.2 (16.7)**	**70.1 (14.0)**	71.0 (12.3)	72.1 (9.3)	72.0 (11.4)	***78.7 (11.2)***
	RoBERTa-para	57.6 (7.6)	57.7 (7.9)	49.0 (2.5)	51.0 (6.3)	54.0 (4.6)	52.0 (9.5)
	GloVe-average	54.8 (6.8)	41.6 (6.2)	64.2 (6.8)	51.0 (4.9)	52.0 (7.9)	61.4 (6.9)
	MPNet-para	63.3 (9.0)	63.3 (11.9)	61.3 (18.1)	62.6 (8.8)	57.6 (10.1)	59.7 (6.9)

*For simplicity, we use the following abbreviations for the pretrained model names: RoBERTa-STS, SYS-RoBERTa-large; RoBERTa-NLI, RoBERTa-large-NLI-mean-tokens; roBERTa-para, paraphrase-distilRoBERTa-base-v2; gloVe-average, average-word-embeddings-gloVe.840B.300d; MPNet-para, paraphrase-MPNet-base-v2. Boldface indicates the highest accuracy for each column in each set of experiments; italic font indicates the highest accuracy overall in each set of experiments*.

### 3.2. Model Validation

In this section, we seek to validate the proposed model against human judgements of warmth and competence. We start by validating the continuous scores assigned by the model (in contrast to the binary label accuracy evaluation above), as compared to real-valued human annotations of warmth and competence. We then further compare the model predictions against survey-based findings reported in the social psychology literature, by analyzing real stereotype data from the StereoSet dataset (Nadeem et al., 2020).

#### 3.2.1. Validation of Real-Valued Scores Against Human Ratings of Warmth and Competence

In Section 3.1, we used the labels provided in the seed lexicon, which for any given word was either +1 or −1 along one dimension, and undefined along the other. However, people associate different seed words with each dimension to various degrees (e.g., *caring* is associated with the warmth dimension more than *sentimental*; *brilliant* is associated with the competence dimension more than *impulsive*). Our computational model also ranks some words higher along each axis than others. Therefore, we wish to evaluate whether the relative rankings of words and sentences agrees with human judgement.

To generate warmth and competence scores manually, we use comparative method of annotation Best-Worst Scaling (BWS) (Louviere and Woodworth, 1990; Louviere et al., 2015). The three authors independently annotated all adjectives associated with sociability/morality (warmth) and ability/agency (competence) in the seed lexicon, in total 235 words. Each word was annotated for both warmth and competence, disregarding their original label in the seed lexicon. The end result of the BWS procedure is a real-valued association score between −1 and +1 for both warmth and competence, for each adjective in the lexicon. The details of the annotation procedure and the annotated data are available in the Supplementary Material.

To generate warmth and competence scores from the model, we embed the annotated adjectives in the test sentence template *These people are always [BLANK]*. We then employ a similar cross-validation procedure as in Section 3.1.3, using 80% of the seed words to generate training sentences, and reserving 20% for testing in each fold. At the end of the cross-validation procedure, we have warmth and competence scores associated with a test sentence for every word in the seed lexicon; we compute Spearman's rank correlation between these scores and the overall BWS annotations. For the sake of comparison, we also train the model using the entire seed lexicon and evaluate the correlation on the full dataset. Table 3 shows that the correlation between manual and automatic scores is within the range of variability between individual human annotators. Therefore, we conclude that the real-valued scores output by the model accurately reflect human judgements of degrees of warmth and competence.

**Table 3:** Correlation between three human annotators (A1, A2, and A3), and between manual and automatic annotations, for warmth and competence scores.

	**Warmth ρ**	**Competence ρ**
Between A1 and A2	0.915	0.884
Between A1 and A3	0.890	0.830
Between A2 and A3	0.852	0.839
Between manual and automatic (cross-validation)	0.870	0.858
Between manual and automatic (full dataset)	0.880	0.873

#### 3.2.2. Validation on Real Stereotype Data

Up to this point, we have relied on synthetic template sentences, such as *These people are always [BLANK]* or *These people are known for being [BLANK] in addition to being [BLANK]*. Our purpose in this section is to validate the model on real text data generated by crowd-workers. Furthermore, we investigate whether we can reproduce findings from the social psychology literature on commonly-held stereotypes and their mapping in the Stereotype Content Model, using the free-text sentence data.

The stereotype data that we use comes from StereoSet, released by Nadeem et al. (2020) for the purpose of measuring stereotype bias in language models. The majority of the dataset is kept hidden as a test set for the project's leaderboard; however, a portion of the data is publicly available as a development set^5^ It is this development set that we use in our present analysis.

The StereoSet data consists of sentences written by crowd-workers on Amazon Mechanical Turk. The workers were given a target group and asked to come up with a sentence that expresses a stereotype about that group. The resulting dataset includes a wide range of target groups, loosely categorized under headings of gender, race/nationality, occupation, and religion. Here, we limit our analysis to those target groups for whom we could identify relevant survey-based measures of warmth and competence in the literature. In many cases the target group labels are not exact matches; we include these groups for completeness but with the caveat that *label content* can itself convey bias, and even when two labels superficially refer to the same social group, they may be associated with different sets of stereotypical associations (Beukeboom and Burgers, 2019). Thus we acknowledge any imperfect matches as a potential source of error. Table 4 shows the target group labels in StereoSet as well as the associated findings from previous research on the Stereotype Content Model.

**Table 4:** Social groups in StereoSet, along with available research findings from the social sciences literature on stereotyping.

**StereoSet label**	**Closest label from literature**	**Prediction**
Nurse	Nurse, (Fiske and Dupree, 2014), Staff nurse (Strinić et al., 2021)	HW-HC
Psychologist	Psychologist (Brambilla et al., 2010)	HW-HC
Researcher	Researcher (Fiske and Dupree, 2014; Strinić et al., 2021)	LW-HC
Commander	Command-and-control leadership (Cuddy et al., 2011)	LW-HC
Manager	Manager (male) (Eckes, 2002), manager (female) (Cuddy et al., 2011)	LW-HC
Entrepreneur	Entrepreneur (Cuddy et al., 2008)	LW-HC
Mathematician, physicist, chemist	Scientist (Fiske and Dupree, 2014)	LW-HC
Engineer	Engineer (Fiske and Dupree, 2014; Strinić et al., 2021)	LW-HC
Software developer	technical experts (Fiske, 2018), tech industry (Lee and Fiske, 2006)	LW-HC
Grandfather	The elderly (Cuddy et al., 2008; Fiske, 2018)	HW-LC
Mommy, Mother	Housewife (Eckes, 2002), Traditional women (Cuddy et al., 2008), working mothers (Cuddy et al., 2004)	HW-LC
Schoolboy, schoolgirl	Children (Fiske, 2018)	HW-LC
Male, gentleman	Men (Glick et al., 2004)	LW-HC
Japanese	Japanese (Lee and Fiske, 2006; Cuddy et al., 2008)	LW-HC
African	African (Fiske, 2018)	LW-LC
Hispanic	Latino (Lee and Fiske, 2006; Fiske, 2018)	LW-LC
Arab	Arab (Fiske et al., 2006)	LW-LC

*The Prediction column lists the expected SCM quadrant for each group, based on the literature. HW, high warmth; LW, low warmth; HC, high competence; LC, low competence*.

For this validation experiment, we obtain the sentence embedding vectors for each stereotype sentence corresponding to each group listed in Table 4, on average 54 sentences per group. In each case, the target group label is removed from the sentence (if it occurs) so that the results are not affected by any bias in the language model regarding that particular group label. We then compute the average of the sentence embeddings and project it onto the SCM plane. We repeat this process using five-fold cross-validation, as above, and report average position over the five folds. Our evaluation metric is *quadrant accuracy*—that is, does the automated method locate the group in the same quadrant of the SCM plane as predicted by the findings of the survey-based literature.

For comparison, we implement the method used by McKee et al. (2021) to compute warmth and competence ratings from free-text responses. Briefly, this involves looking up each word in a given text in the extended lexicon provided by Nicolas et al. (2021); if the word is positively associated with warmth then the warmth score is increased by one, and if it is negatively associated with warmth then the warmth score is decreased by one (and similarly for competence). Then the scores for each dimension are normalized by the length of the text. Here, we consider each StereoSet sentence to be a separate text. We compute the warmth and competence scores for each sentence, and then average over all sentences for a given target group. For each dimension, if a sentence contains zero words associated (positively or negatively) with that dimension, then the sentence is simply not included in the computation.

**Figure 1:**
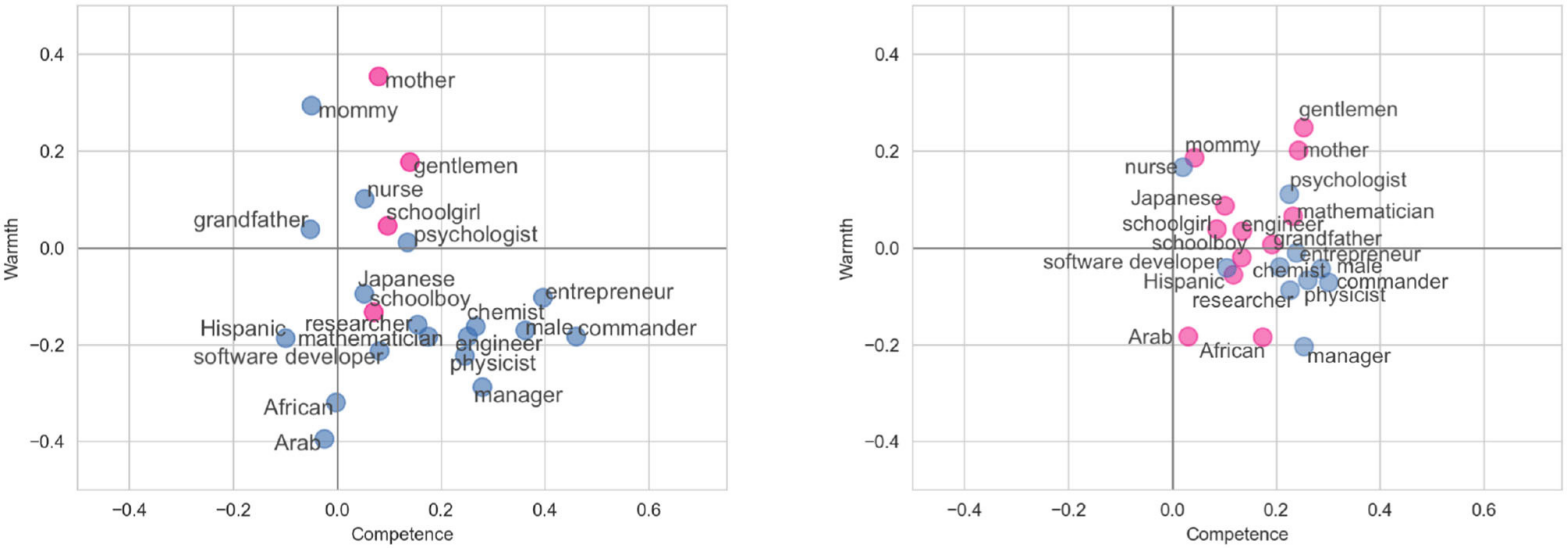
**(Left)** Plotting the average of the StereoSet stereotypes using the proposed method. **(Right)** Plotting the stereotypes using the baseline method of McKee et al. (2021). Groups which are correctly categorized according to the predictions of the literature are shown in blue, while those which are incorrectly categorized are marked with pink.

The results of the experiment for our proposed method and the baseline method of McKee et al. (2021) can be seen in Figure 1. Our proposed methodology leads to a quadrant accuracy of 82%, compared to 45% for the baseline method. Manual examination of the cases where our SCM model makes a prediction that is not congruent with the previous literature shows that the differences are mostly due to the mismatches in the target group names (e.g., *gentlemen* vs. *male*) and, as a result, in associated stereotypes. A qualitative comparison of the methods reveals several potential benefits of the proposed sentence-embedding approach in contrast to the baseline method:

- **Broader coverage:** Most of the sentences did not contain any words from the extended lexicon, leading to a data sparsity problem (63% of sentences did not contain a word associated with competence in the lexicon, and 60% did not contain a word associated with warmth in the lexicon). The sentence embedding method ensures that a score exists for every sentence.
- **Context sensitive:** As demonstrated in Table 2, the RoBERTa sentence embeddings are able to handle important contextual information such as negation, which is not possible in the baseline method. For example, the baseline method assigns a positive competence score to the sentence *They are poorly educated and prone to criminal behavior* due to the presence of the word *educated*; the proposed method correctly assigns this sentence a negative competence score.
- **Word-sense disambiguation:** The baseline method is not able to distinguish when words are being used in a different sense from the extended lexicon. For example, in the sentence *He holds a beaker in his hand and looks like an evil scientist*, the word *like* is associated with positive sociability and the sentence is scored overall with positive warmth. Our method scores the sentence as expressing negative warmth.

To sum up, in this section we have presented a sentence-level model of warmth and competence, combining the seed words from Nicolas et al. (2021) with sentence templates and pretrained sentence embeddings. We proposed two refinements to the model: (1) a change to the training paradigm, which allowed us to use sentences which were well-defined in terms of both warmth and competence, and (2) an intermediate dimensionality reduction step using PLS. We then evaluated the model across various sentence embedding models, taking into account different linguistic structures we wanted the model to be capable of handling. Finally, we validated the best-performing model against manually-annotated real-valued ratings of words from the seed lexicon, as well as using crowd-sourced sentences expressing stereotypes.

## 4. Case Studies

We now deploy our model in a more exploratory setting, to demonstrate how it might be used in practice to analyze stereotypical language “in-the-wild.” We present the results of two preliminary case studies of Twitter data: in the first, we examine perceptions of women in general, as well as certain sub-categories of women, and in the second, we explore how different category labels for older adults (e.g., *senior citizens* vs. *old people*) are associated with differing expressions of warmth and competence.

Twitter represents, to use the vocabulary of Goldstone and Lupyan (2016), a “naturally occurring dataset” for psychological research. It is a rich source of real-time opinions and commentary from a massive user base, varying in age, sex, location, socioeconomic status, and education level. We believe that these characteristics make it a potentially interesting data source for studying stereotypes. However, using Twitter data also introduces some challenges. In particular, we do not expect that every post on Twitter expresses a stereotype or generalization. Many Twitter posts are factual statements about the world (e.g., news headlines), or descriptions of a particular person or event. In the following sections, we describe in detail the steps we take to focus specifically on (1) generalized beliefs about particular social groups, and (2) evaluations of warmth and/or competence that are expressed in a large proportion of our collected data, as opposed to isolated opinions of individual users. Finally, we emphasize that the techniques we employ in this section could equivalently be applied to other text sources, including books, TV and movie dialogues, personal essays, blogs, or any other textual data of interest.

### 4.1. Data and Methods

**Figure 2:**
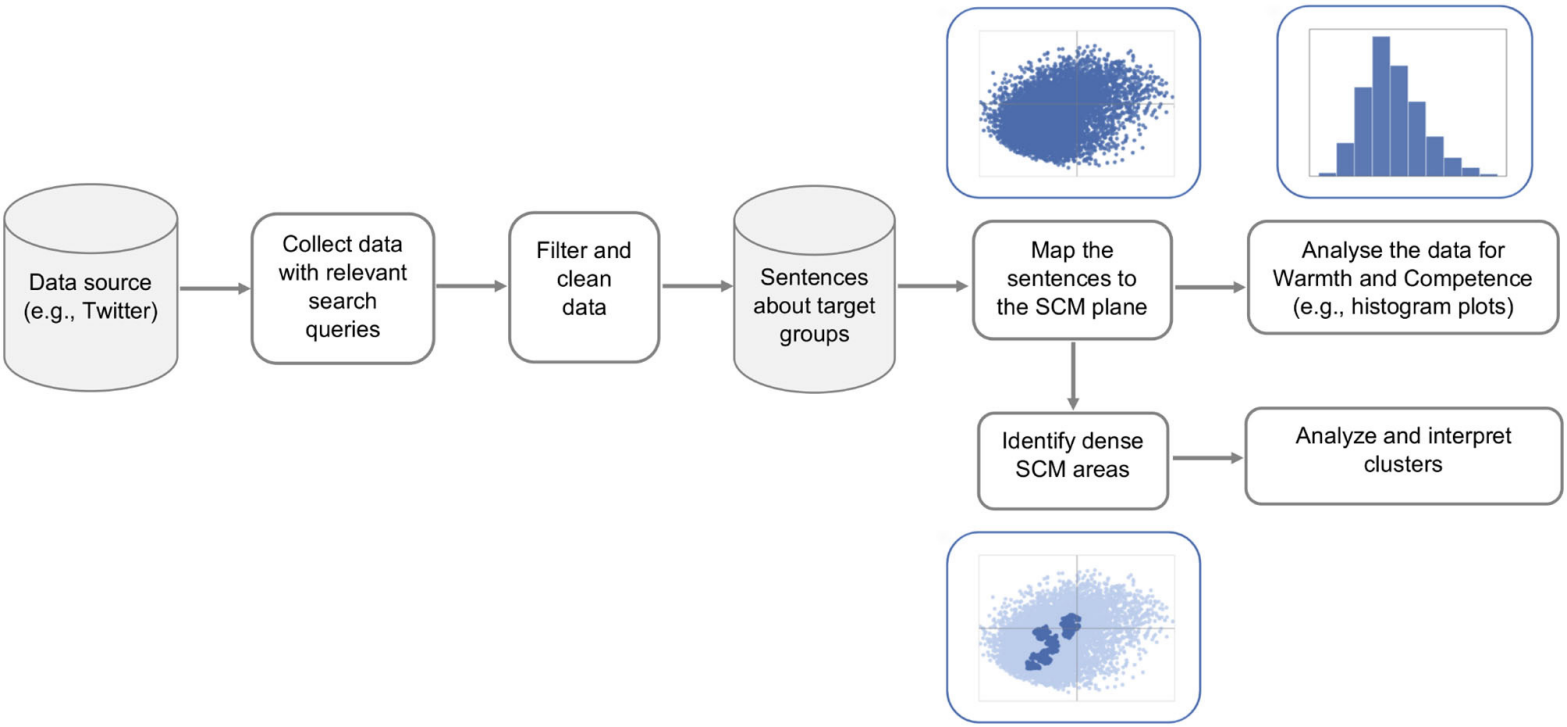
Analyzing stereotypical language “in-the-wild”: steps for collecting, filtering, and analyzing real-life data about social groups.

The overall process for collecting and analyzing real-life data for potential stereotypes is depicted in Figure 2. Here, we start by collecting tweets using the Twitter API with query terms representing target groups of interest. Not all tweets mentioning the target group actually state generalized opinions about the group. This is especially true for more frequently used terms, for example, *women*. This word can refer to the general group of women, but can also be used to talk about women's soccer team, women's fashion, certain women politicians, etc. Therefore, we focus on sentences with specific syntactic structures, where the term representing the target group is the nominal subject in the main or a subordinate clause of the sentence. Studies in psychology reveal that stereotypical associations are often expressed through the use of abstract terms, such as adjectives (Maass, 1999; Ellemers, 2018). So, for large datasets, in our case, for women-related groups, we apply an even more restrictive syntactic pattern “ <target group> are <adjective>.” We notice that although filtering based on this syntactic pattern excludes a lot of relevant sentences, it returns relatively high-quality data. We use the spaCy library^6^ to separate tweets into sentences and perform dependency parsing for each sentence. We further discard sentences where the target group is described with qualifiers that refer only to some members of the group (e.g., *some, these, many*, etc.).

The extracted sentences are further cleaned by removing URLs (for embedded images and videos) and user mentions. We also mask the words indicating the target group in each sentence to avoid possible bias for or against the group that might be present in the sentence embedding model. Then, we map each sentence to its 1,024-dimensional RoBERTa representation and apply our computational SCM model to project the sentences onto the two-dimensional SCM plane. We analyze the projections for each target group and compare the groups in terms of their score distributions for warmth and competence.

To identify regions of interest in the warmth-competence plane, we employ a clustering method called HDBSCAN, developed by Campello et al. (2013). It is a hierarchical, density-based clustering algorithm, which works by finding areas of high-density in the input space, and discarding points in low-density areas as outliers. This method is well-suited to our particular task, since by definition we want to find widely-held and commonly-repeated ideas about our target group, and to ignore statements which merely express an individual's idiosyncratic opinion.

Since each cluster contains hundreds or thousands of sentences, we use an automated method to help interpret the clusters by extracting words that tend to appear in a particular cluster, but not in the others. We perform this analysis using Pointwise Mutual Information (PMI). We choose the PMI method due to its simplicity and robustness, and it has been successfully applied in a number of similar NLP contexts (Kiritchenko et al., 2014; Clark et al., 2016; Rudinger et al., 2017). However, we note that other methods to estimate the degree of association of a word with a category (e.g., cross entropy, Chi-squared test, and information gain) can be used instead.

Additional details of the Twitter data collection and pre-processing, HDBSCAN parameter-tuning, and the PMI method can be found in the Supplementary Material.

### 4.2. Case Study 1: Uncovering Sub-stereotypes of Women

We first explore how the model can be used to analyze perceptions about women expressed on Twitter. Gender stereotypes have been extensively studied and we provide only a brief discussion here; see Ellemers (2018) for a recent review. We focus here on stereotypes of women, although stereotypes of men have also been studied (Glick et al., 2004), and emerging research has begun to examine the stigmas attached to nonbinary and genderqueer people (Worthen, 2021).

Women are often stereotyped directly in contrast to men, specifically that women are seen as warmer, more family-oriented, more social, less competent, and less ambitious than men (Ellemers, 2018). However, the group of all women is large and varied, and as a result, it is likely that one will encounter a woman who does not fit the generalized stereotype. When this happens, rather than abandoning the stereotype, often the perceiver will instead maintain the stereotype and assign the “exceptional” individual to a new category through the process of *subtyping*. In other cases, rather than excluding the exceptions from the boundaries of the superordinate group, perceivers will create new sub-groups under the umbrella of the superordinate group, in a process known as *subgrouping* (Richards and Hewstone, 2001). There are multiple theories of how women are typically subcategorized. Glick and Fiske (1996) proposed the Ambivalent Sexism Inventory, introducing the categories of *hostile sexism* (antipathy toward women) and *benevolent sexism* (marked by paternalism, gender differentiation, and ideals of heterosexual intimacy). Subsequently, Glick et al. (1997) showed that men who exhibit ambivalent sexism (as compared with men who do not exhibit sexism) tend to categorize women into polarized subgroups of “good” and “bad,” which allows them to resolve the complexity of an ambivalent perception of women as a large group of people. They also analyzed the hostile and the benevolent components of the ambivalent sexism separately, and found that hostile sexism is related to evaluation of women in a nontraditional role (career women), whereas the positive component evaluates women in traditional roles (homemakers).

The Stereotype Content Model further develops the theory of ambivalent sexism with reference to the warmth and competence dimensions. In this model, benevolent sexist beliefs are associated with high warmth and low competence and hostile sexist beliefs are associated with high competence and low warmth (Cuddy et al., 2008). Eckes (2002) reported clusters of women following this pattern, with women like “housewives” and “secretaries” embodying the benevolent sexist stereotype of women as warm but not competent, and women like “career women” and “feminists” seen as competent but cold. However, in contrast to this finding, Wade and Brewer (2006) found that subcategories of women were distinguished along the dimension of valence, rather than warmth or competence, suggesting that ambivalent stereotypes create contradictory feelings of liking and disliking, which is often resolved by isolating subgroups that are fully likable or completely unlikable.

Furthermore, people can be stereotyped at the intersection of gender and other social categories. Studies have found that the content of intersectional stereotypes—for example, stereotypes of Black women—contain elements that do not result from simply adding together stereotypes of Black people and stereotypes of women (Ghavami and Peplau, 2013). Landrine (1985) found that Black women were more likely to be stereotyped as *dirty, hostile*, and *superstitious*, while white women were stereotyped as *dependent, emotional*, and *passive*, although both groups were also jointly viewed as stereotypically feminine (i.e., less intelligent, capable, and ambitious than men).

Here, we examine how women and labeled subgroups of women are described by Twitter users, which subgroups are portrayed as warmer or more competent, and which words are most highly associated with which groups.

#### 4.2.1. Results

Based on previous studies of gender stereotypes, we selected the following query terms: *women, moms, feminists, businesswomen*, and *housewives*. Using these query terms, over 14 million tweets were collected. We noticed that query terms *businesswomen* and *housewives* are rarely used on Twitter, and the latter is mentioned primarily in relation to TV series “Desperate Housewives” and “Real Housewives.” Therefore, we decided to focus on the other three terms: *women, moms*, and *feminists*. After filtering the data as described in Supplementary Materials, we were left with 3,563,605 tweets written by 1,610,667 unique Twitter users.

From these tweets, we extract sentences using the syntactic pattern “ <target group> are <adjective>.” We discard sentences with the word *men* to avoid situations where men and women are discussed together. We observe that many of the sentences about *women* actually refer to specific subgroups of women (e.g., Asian women, young women, pregnant women, etc.). We select two of these subgroups, *Black women* and *white women* for further study, as they represent groups with distinct, contrasting stereotypes that are often discussed in relation to controversial topics, such as race and discrimination. Then, to minimize the influence of any other specific sub-groups, for the superordinate group *women* we only select sentences where women are referred to without any modifiers. Thus, in total, we analyze five target groups: the superordinate group *women* and four subordinate groups, *feminists, moms, Black women*, and *white women*. Table 5 presents the statistics on the extracted sentences for our target groups of interest.

**Table 5:** The number of extracted sentences with the syntactic pattern “ <target group> are <adjective>” and the average and total number of words (sequences of alpha-numeric characters) in the sentences for each women-related target group.

**Target group**	**Number of**	**Avg. number of**	**Total number**
	**sentences**	**words per sentence**	**of words**
Women	28,229	12.96	365,911
Feminists	862	14.85	12,804
Moms	1,906	10.04	19,135
Black women	2,423	12.69	30,737
White women	1,000	12.52	12,522

We begin by calculating the overall distributions of warmth and competence values for our generic *women* category and the subcategories of interest (see the Supplementary Materials for data visualizations). We observe that Black women and moms are described as more competent than women in general, and white women and feminists are described as less competent. Similarly, Black women and moms are described as warmer than white women and feminists.

**Figure 3:**
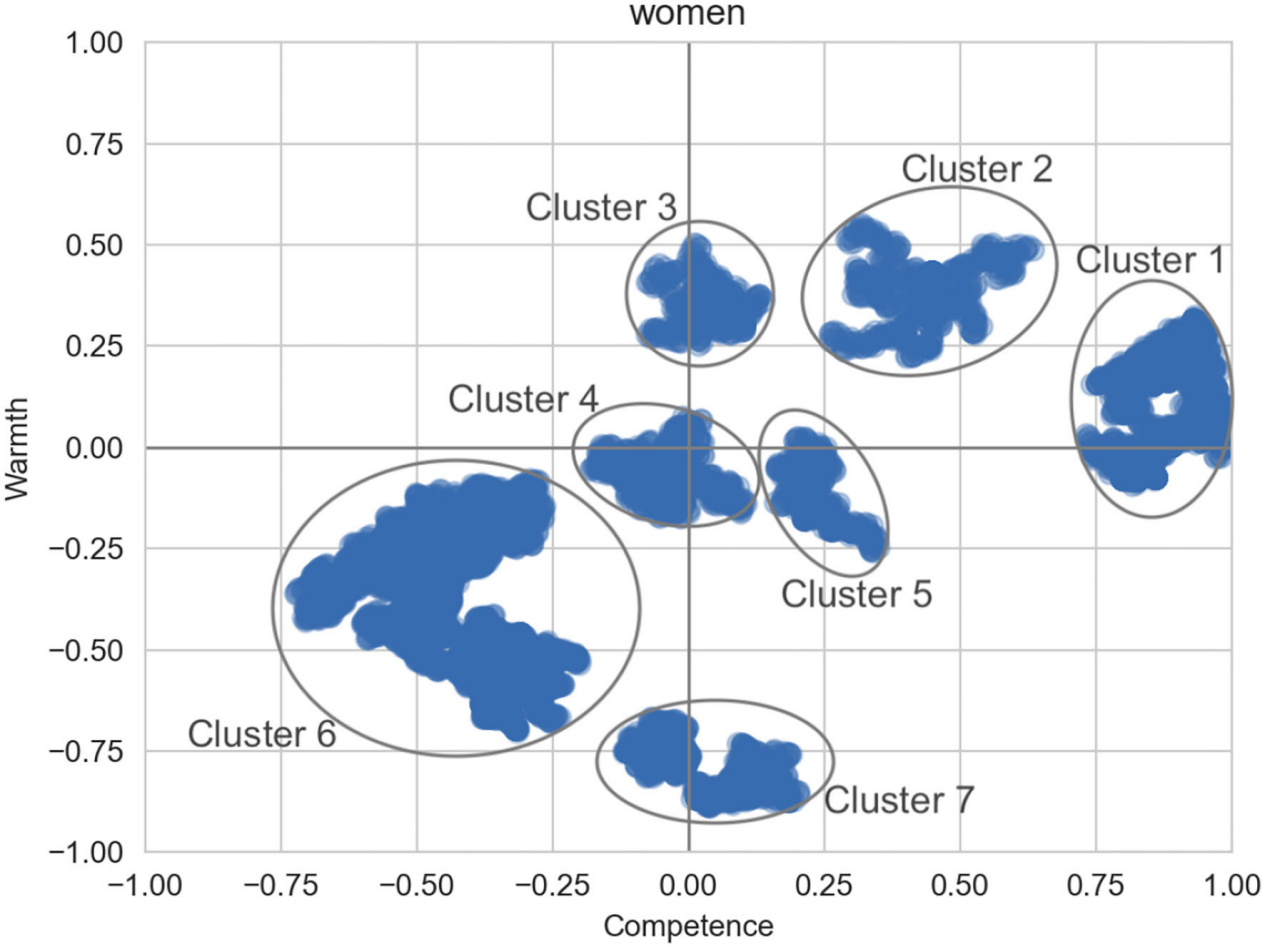
Areas of highest density for the group *women*.

We then perform the cluster analysis. Figure 3 shows the highest density clusters for the general women group. The words associated with each cluster in Figure 3 can be seen in Table 6, along with examples of the words in context (These examples are paraphrased rather than exact quotes, to preserve user privacy). Cluster 1 is very high competence, although examination of the salient words indicates that one alleged area of competence for women is being *beautiful* and *hot*. However, we also observe statements that women are *powerful* and *strong*, in line with traditional views of competence. In the same vein, Cluster 2 contains beliefs about women's autonomy and ability to tackle challenges.

**Table 6:** Words associated with different clusters and paraphrased example contexts where the words appear for each women target group.

**Cluster**	**No. of sent**.	**Cluster location**	**Words associated with cluster**	**Paraphrased example contexts**
**Women**				
Cluster 1	1,886	*W* ^+^ *C* ^+^	*Amazing, superior, strong, stronger, powerful, beautiful, best, better, hot, pretty*	*Women are amazing, strong, stronger than ever, powerful, beautiful, hot, capable of making their own decisions*
Cluster 2	475	*W* ^+^ *C* ^+^	*Cool, love, human, pretty, free, right, beautiful, capable, real*	*Women are free with their choices, are always right, are capable of doing any job, are fully human and have human rights, I love seeing women successful*
Cluster 3	305	*W* ^+^ *C* ^0^	*Free, happy, taliban, love, safe, able*	*Women are free to make their choices, are safe under the new Taliban rule, are in love with compliments*
Cluster 4	503	*W* ^0^ *C* ^0^	*Trans, female, need, male*	*Women are biologically female, trans women are biologically male, women are diverse in their needs*
Cluster 5	314	*W* ^0^ *C* ^+^	*Smarter, hot, amazing, pretty, better, male, strong*	*Women are smarter in politics, better at intuitive thinking, amazing, but nonsensical, strong in a way different from males*
Cluster 6	3,117	*W* ^−^ *C* ^−^	*People, woman, funny*	*Some people think that women are weak and inferior, many people believe women are not funny*
Cluster 7	1,150	*W* ^−^ *C* ^0^	*Wicked, evil, mad, angry, safe*	*Women are wicked, evil, mad, are not safe all over the world, are angry because their rights are violated*
**Feminists**				
Cluster 1	47	*W* ^+^ *C* ^+^	*Inclusive, fine, happy, right, trans*	*Feminists are right on many issues, are inclusive, support transgender people's rights*
Cluster 2	386	*W* ^−^ *C* ^−^	*Male, white, people, silent*	*Male feminists harass women, white feminists are racists, feminists are silent on various issues*
**Moms**				
Cluster 1	444	*W* ^+^ *C* ^+^	*Awesome, best, cute, great, fun, amazing, right, good*	*Moms are awesome, fun, always right, are the best*
Cluster 2	140	*W* ^+^ *C* ^0^	*Young, friend, mom, day, time, need, hot, kids, right*	*Moms are hot, protective of their young kids, manage to do many things in a day*
Cluster 3	434	*W* ^−^ *C* ^0^	*Worst, worse, weird, toxic, scary, mad, think, bad, boy*	*Deadbeat moms are the worst, toxic moms are worse than absent dads, moms are scary when mad, boy moms are over-protective of their sons*
**Black women**				
Cluster 1	742	*W* ^+^ *C* ^+^	*Amazing, undefeated, beautiful, gorgeous, elite, fine, damn, truly*	*Black women are truly amazing, undefeated, so damn beautiful, black women are elite*
Cluster 2	188	*W* ^0^ *C* ^+^	*Educated, refocus, funny, superior, thick, country, best, wear, elite, better*	*Black women are the most educated demographic in the US, black women are strong, funny, superior to other groups*
Cluster 3	647	*W* ^−^ *C* ^−^	*Die, pregnancy, childbirth, ugly, white, tired, people*	*Black women are more likely to die from childbirth or pregnancy related issues than white women, are tired of having to be strong, it's preposterous that some people think black women are ugly*
**White women**				
Cluster 1	39	*W* ^0^ *C* ^+^	–	–
Cluster 2	112	*W* ^0^ *C* ^0^	*Different, comfortable, re-publican, human, good, racism, funny, attractive, complicit, abortion*	*White women are funny to watch, more likely to afford abortion, complicit in white supremacy, comfortable insulting people*
Cluster 3	520	*W* ^−^ *C* ^−^	*Worst, bad, weird, dangerous, worse, liberal, evil*	*White women are entitled and dangerous, are evil, white liberal women are the worst racists*

*Up to 10 words with highest association with the cluster are shown. The cluster locations on the warmth(W)–competence(C) plane are denoted as ^+^(pos), ^−^(neg), ^0^(neutral)*.

Cluster 3 is the highest-warmth cluster, although it is not particularly warm and does not address traditional warmth-based stereotypes of caregiving and motherhood, but rather focuses on generically positive words like *love* and *happy*. Clusters 4 and 5 are relatively neutral in terms of both warmth and competence, and involve comments about women's biology and social roles.

Cluster 6 is in the low-low quadrant, but generally lower on competence than warmth. Interestingly, many sentences in this cluster appear to report “other people's” negative opinions of women (for example, that they are inferior, or not funny). Finally, cluster 7 is extremely low on warmth. It contains a mix of ideas, some suggesting that women are simply terrible (wicked, evil, etc.), while in other cases providing justification for why women might exhibit low-warmth characteristics, such as anger at an unjust situation.

**Figure 4:**
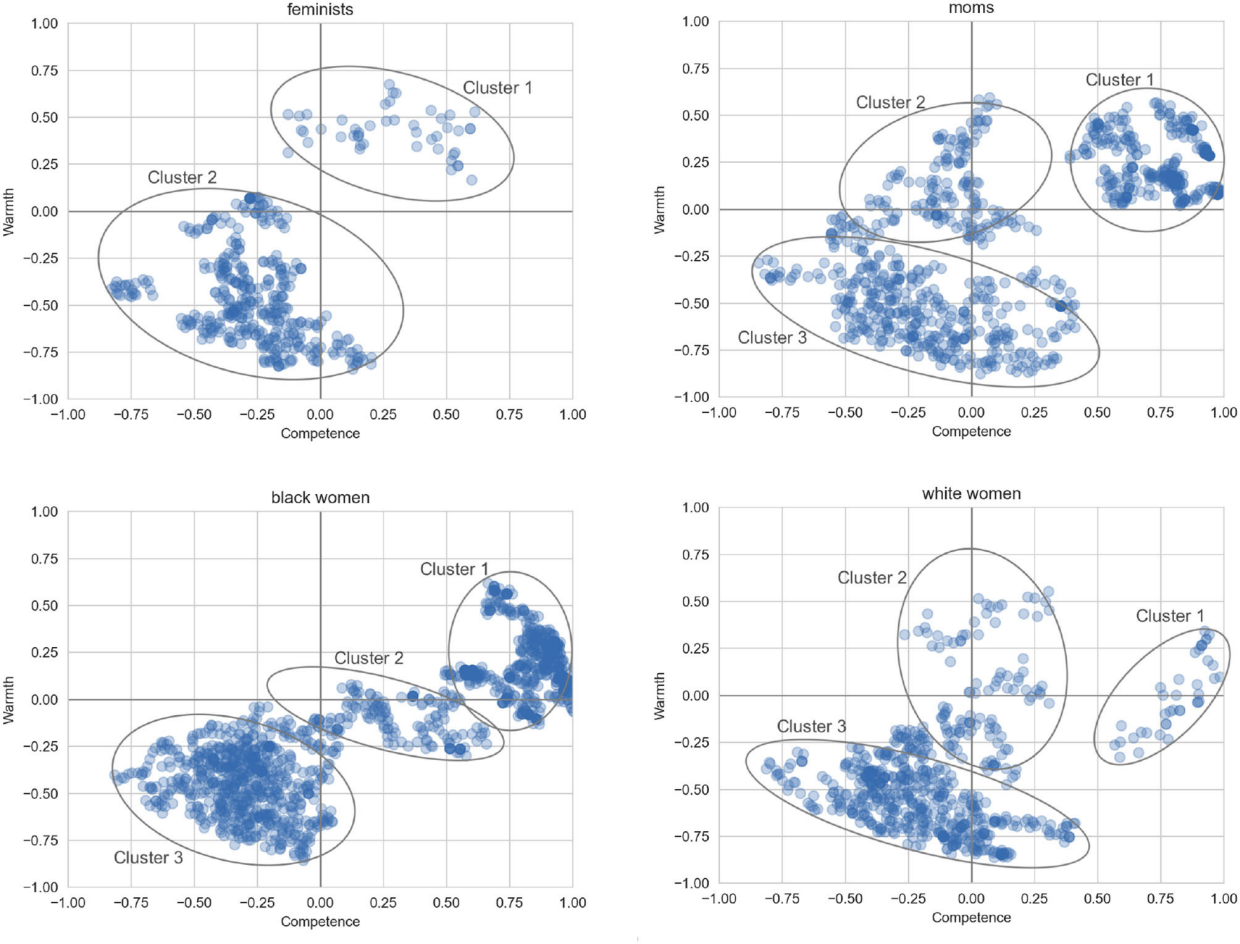
Areas of highest density for the different groups of women.

In comparison, the clustering results for the four sub-categories are given in Figure 4. The cluster boundaries are not as well-defined due to the smaller dataset size compared to women in general. However, we can observe some similarities and differences.

In the case of feminists, there is a large cluster in the low-low quadrant, and a smaller cluster mostly lying within the high-high quadrant. From Table 6, the positive cluster focuses on the achievements of feminists toward the pursuit of a more just and equitable world. The second, larger cluster derogates feminists and in particular seems to focus negatively on subgroups such as male feminists and white feminists.

The plot for moms is characterized by three clusters. There is a large positive-competence cluster (1), espousing the view that moms are “the best.” Cluster 2 is less competent but still warm, and includes beliefs about moms being protective of their children and having to do many things in a day. The last cluster covers a range of competence values but all points have negative warmth, describing moms as *scary, mad*, and *toxic*.

The cluster analysis for Black women also results in three clusters. Cluster 1 again represents high-competence views, with Black women described as *amazing, beautiful*, and *elite*. Cluster 2 is also high-competence, although crossing into the negative warmth quadrant, discussing the educational attainments and claiming the superiority of Black women. Cluster 3 is in the low-low quadrant, although the words and contexts suggest that many sentences could be interpreted as supportive, for example, drawing attention to healthcare disparities and mental health issues that can be experienced by Black women.

Finally, the data for white women also falls into three main clusters. The PMI analysis for the high-competence cluster does not return any highly-salient words, although an examination of the data reveals opinions along the lines that white women are *beautiful* and *attractive*. Cluster 2 is relatively neutral on competence and covers a range of warmth assessments on topics such as abortion and white supremacy. Cluster 3 lies mostly within the low-low quadrant and contains allegations that white women are *dangerous, entitled*, and *racist*.

#### 4.2.2. Discussion

We conclude our first case study with a few high-level observations about the results, as well a discussion of the benefits and limitations of the method.

**Comparison of Superordinate and Subordinate Groups**

The superordinate group *women*, as well as three out of four subcategories of women (*feminists* being the exception) all included a cluster high along the competence axis. While this cluster often contained assessments like *powerful* and *strong*, it also typically involved references to women's beauty. The fact that mentions of physical appearance should be rated so high on the competence dimension by our computational model would seem to reflect underlying bias in the sentence embedding model. However, this bias is also reflected in research findings, suggesting that people do in fact correlate physical attractiveness with social and intellectual competence (Eagly et al., 1991). The fact that feminists are the only subgroup of women *not* characterized by their physical beauty is, perhaps, stereotypical in and of itself.

We also observe a sizeable cluster in the low-low quadrant for every subcategory of women, as well as women in general. However, the PMI analysis reveals that the content of this cluster differs across groups. For example, the women superordinate category contains negative statements about women, as well as descriptions of women's reactions to their negative circumstances. This distinction is mirrored in the negative clusters for white women (assigned negative traits directly) and Black women (assigned situation-dependent negative traits). This is consistent with the definition of sub-grouping in Richards and Hewstone (2001): here we see that both Black and white women are still assigned the stereotypical traits of women more generally (thus they do not disconfirm the general stereotype of the superordinate category), but they are distinguished in other ways.

**Comparison With Survey-Based Approaches**

The clusters in Figures 3, 4 are noteworthy in their discrepancy with previous, survey-based studies predicting ambivalent gender stereotypes. We suggest that a number of factors contributing to this result.

First, the experimental design of capturing naturally-occurring data from Twitter is clearly quite different from a well-controlled laboratory study. Aside from the obvious point that we are not directly eliciting stereotypes, we also have the added issues of audience and social desirability factors. In the survey-based studies, individual responses are not made public, and the investigators were careful to mitigate social desirability concerns by stating “We are not interested in your personal beliefs, but in how you think they are viewed by others” (Fiske et al., 2002). Here, we are limited by what users choose to reveal publicly on Twitter. The benefit, though, is that we receive directly the spontaneously expressed opinions of individuals, rather than their secondhand knowledge of broad cultural stereotypes.

In a related issue, the fact that we do not know whose opinions we are capturing blurs the line between in-group and out-group. While previous studies suggest that many gender stereotypes are held similarly by both men and women (Heilman, 2012), other work has also shown that, for example, men rated women as having lower agency than men (Hentschel et al., 2019). When we consider the subcategories, it seems even more likely that people who self-identify as feminists (an aspect of identity which is chosen rather than assigned) will have different views of feminists than those who do not consider themselves part of that group. Therefore, our results present a cross-sectional view of Twitter users in general, rather than of a particular cultural or demographic group.

Furthermore, and again unlike the survey-based methods, the Twitter users are not presented with target group labels; they are choosing themselves what groups they want to talk about and how to label them. This introduces interesting contrasts in terms of what is said and unsaid; marked and unmarked. What motivates people to post about something on Twitter? Brekhus (1998) discusses in depth the “sociology of the marked” —people are generally most interested in studying and talking about phenomena which are exotic or extreme, while the mundane and typical are not considered worthy of comment. Here, however, this avoidance of commenting on the typical might bias our search to uncover the stereotypical. Furthermore, unmarked items are taken to be the typical cultural default. This is relevant in the discussion of white women vs. Black women, since in North American society, white normativity implies that when race is not specified, the default assumption is whiteness. So when people write *white women*, they are deliberately drawing attention to race, often as an explicit reference to white people's racial privilege. Therefore it is perhaps unsurprising that many of the expressed perceptions of white women are quite negative, referencing the stereotype of white women as entitled and racist.

Another motivation for posting on Twitter is its popularity as a platform for political and social justice movements. Carney (2016) describes Twitter as a new public sphere, accessible to people who were, for various reasons, previously excluded from the public discourse. She describes specifically how the #BlackLivesMatter movement demonstrated how “youth of color challenge dominant ideologies of race through social media.” Other researchers have analyzed how individuals and organizations use Twitter to produce *counterspeech*, or speech which actively aims to dispute and de-legitimize abusive or hateful comments online (Wright et al., 2017; Mathew et al., 2018). Therefore, we propose that some of the opinions uncovered in this analysis might actually represent counter-stereotypes (e.g., *women are stronger than ever* fights the “weak, helpless woman” stereotype; *Black women are highly educated* fights the “welfare queen” stereotype).

Finally, we note the relative scarcity of ambivalent stereotypes, as predicted by the SCM. This may be partly due to our clustering procedure: prior to clustering, we do observe many sentences mapped to the ambivalent quadrants, but they are not selected as high-density areas of interest. Research has indicated that social media can have an “echo chamber” effect, leading to highly polarized views being propagated through the network, and more nuanced opinions becoming less popular (Prasetya and Murata, 2020). This polarizing effect may contribute to our results. Additionally, we note that analysis of a single sentence offers only one perspective on the speaker's overall view of a social group. Their complete cognitive representation of a group may include many additional associations which are not referenced in this particular sentence.

### 4.3. Case Study 2: Comparing Age-Related Stereotypes

In the previous case study, we examined how a single group maybe be sub-categorized and stereotyped in different ways. Here, we explore a different aspect of stereotyping: how the *label* used to describe a group communicates bias, through both form and content.

Beukeboom and Burgers (2019) propose the Social Categories and Stereotypes Communication (SCSC) framework to explain the role of language in the communication of stereotypes. In particular, they describe how biases can be encoded in the labels used for social groups, in terms of label content and the linguistic structure of the label. Beyond mere descriptors, labels can convey additional associations about a group: consider for example the different connotations between *immigrants, refugees*, or *aliens*. Beukeboom and Burgers describe the relationship between label content and stereotype content as “two-directional,” since the category label can activate certain stereotypical associations, but also, speakers who hold particular stereotypical views are more likely to use certain labels. The linguistic *form* used to label groups is also a meaningful aspect of stereotype communication. When an adjective is used to describe a person's membership in a group (e.g., *he is Jewish*), it is seen as just one aspect of the person's identity. However, when a noun phrase is used instead (e.g., *he is a Jew*), it can imply that this is an essential and immutable aspect of this person, and makes it harder to envision the person as belonging to alternative social categories. Here, we examine whether four different labels used to refer to older adults—the *elderly, senior citizens, old folks*, and *old people*—are associated with differing stereotype content.

Older people are often stigmatized in today's society. Research shows that age-related stereotypes exist and are expressed even by children as young as 3-years-old (Flamion et al., 2020). North and Fiske (2013) discuss the harmful social effects of *prescriptive stereotypes* of older adults, which focus on expectations of how older adults allegedly “should” behave. When these expectations are violated, it elicits feelings of anger and resentment, particularly amongst young people. Furthermore, such stereotypes can become a self-fulfilling prophecy when they are internalized by people who self-identify as older adults, leading to isolation and health decline (Chan et al., 2020).

Blaine and Brenchley (2017) reviewed stereotypes associated with sub-groups of older people. They explained that the superordinate group of “all old people” is often stereotyped based on physical traits such as gray-haired, hard of hearing, and poor eyesight, reflecting low levels of competence but at the same time high levels of warmth. In contrast, sub-groups might be stereotyped as fully negative, such as the “shrew/curmudgeon” stereotype of being ill-tempered and nosy, or fully positive, such as “perfect grandparent” seen as kind family-oriented, and wise (Hummert et al., 1994; Cuddy and Fiske, 2002; Blaine and Brenchley, 2017).

The COVID-19 pandemic has led to a proliferation and reinforcement of certain stereotypes of older adults. Fraser et al. (2020) describe how the public discourse around the pandemic frames older adults as frail, vulnerable, and in the worst case, less valuable members of society than younger people. Lichtenstein (2021) report how the media in three English-speaking countries portrayed older adults as needing protection and isolation, or alternatively suggesting that older adults should be willingly sacrificed in the pursuit of herd immunity. While varying in terms of warmth, both of these views convey an impression of low-competence. Berridge and Hooyman (2020) describe how pandemic recommendations referring to all adults over the age of 60 as a homogeneous group, using words like *seniors* or *the elderly*, can promote paternalistic stereotypes, as well as sow confusion.

Here, we enumerate a set of terms that refer to older adults, and ask whether these terms correspond to specific types of stereotypes that Twitter users might hold about older people.

#### 4.3.1. Results

We collected tweets using the following query words: *elderly, elderly people, elderly folks, elderly persons, old people, old folks, old persons*, and *senior citizens*. Close to 720K tweets were obtained. Based on the number of available tweets, we decided to focus on four, most frequently mentioned groups: *elderly, old people, old folks*, and *senior citizens*. After filtering, there are 205,897 tweets written by 157,107 unique Twitter users for the four groups. The overall number of collected tweets is significantly lower than for the women data collection, and so we extract sentences with a less restrictive syntactic pattern, only requiring for the target group to be the nominal subject of the main or subordinate clause. Table 7 shows the numbers of the extracted sentences for these four target groups.

**Table 7:** The number of extracted sentences with the target as nominal subject and the average and total number of words (sequences of alpha-numeric characters) in the sentences for each age-related target group.

**Target group**	**Number of**	**Avg. number of**	**Total number**
	**sentences**	**words per sentence**	**of words**
Elderly	7,840	19.40	152,097
Old folks	2,126	16.03	34,076
Old people	19,812	15.07	298,499
Senior citizens	1,705	17.46	29,766

When we compute the overall distributions of warmth and competence (see Supplementary Material), we observe that all four groups are similarly ranked on competence, with *elderly* having a slightly lower mean than the others. In terms of warmth, the distributions are again similar, with *senior citizens* appearing to be slightly warmer in general, and *elderly* slightly colder.

**Figure 5:**
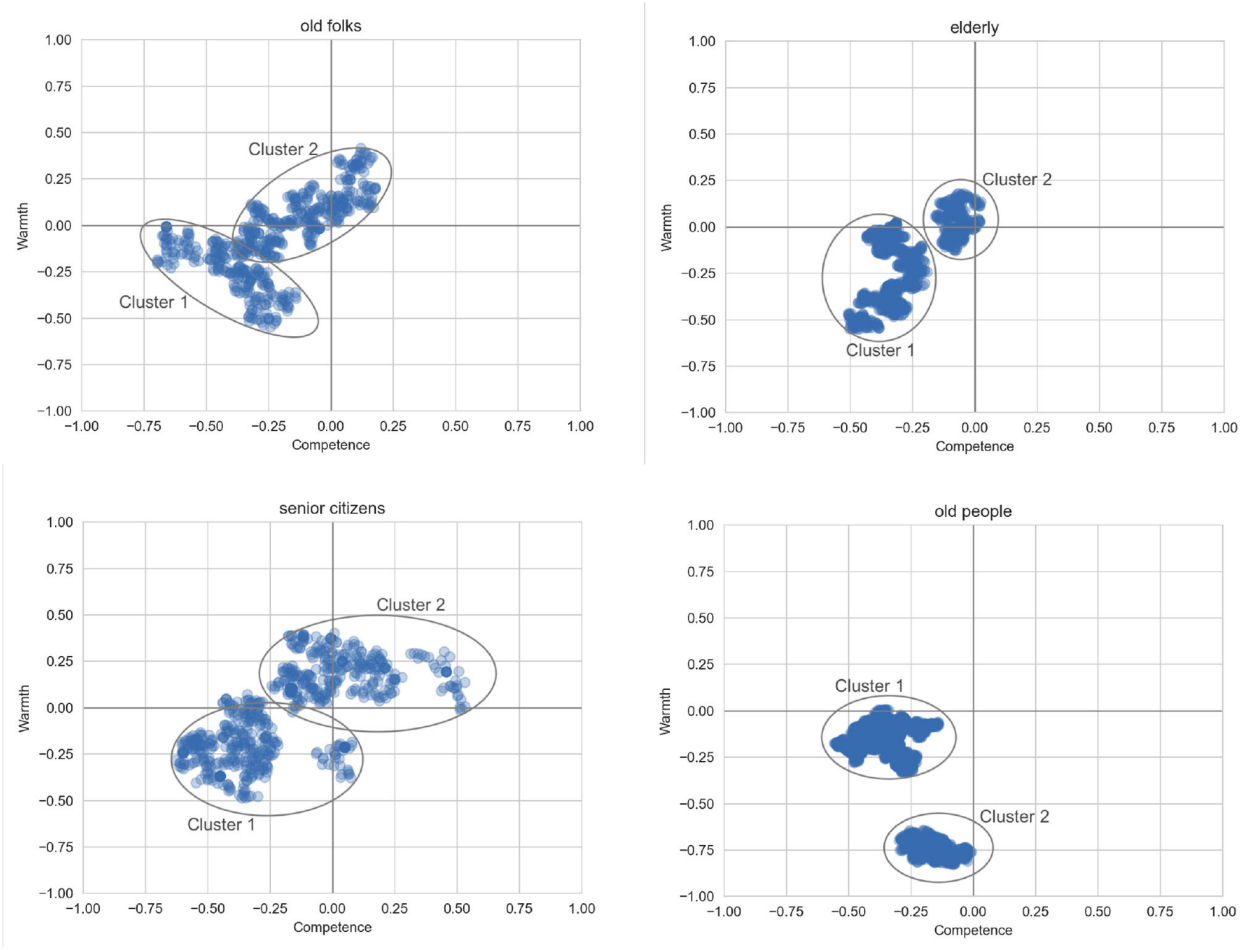
Areas of highest density for the different labels for older adults.

To examine the most densely populated areas of the 2D plane, we again use HDBSCAN to cluster the datapoints. The clustering results are given in Figure 5. We observe that all four groups have one cluster corresponding to low-competence and moderately low-warmth. The PMI analysis (Table 8) indicates that these clusters are dominated by statements about COVID-19 and its negative effects on older adults. For *old folks*, the second cluster is quite neutral, and discusses relatively innocuous topics such as how old folks like to go to bed early. For the *elderly*, the second cluster is also quite neutral in terms of warmth and competence, although very different in content as it discusses the need for the elderly to be vaccinated and be given social care. The second cluster for *senior citizens* is more positive than the other three cases, with some sentences suggesting that senior citizens should be honored and respected. In contrast, the second cluster for *old people* is strongly negative, suggesting that old people are *rude, annoying*, and *judgemental*.

**Table 8:** Words associated with different clusters and paraphrased example contexts where the words appear for each age-related target group.

**Cluster**	**No. of sent**.	**Cluster location**	**Words associated with cluster**	**Paraphrased example contexts**
**Elderly**				
Cluster 1	1,440	*W* ^−^ *C* ^−^	*Deaths, die, died, death, COVID, kids, help, dying, children, homes, vulnerable*	*Elderly make up majority of COVID-related deaths, are most vulnerable, why do kids need vaccination if elderly are the ones at risk, elderly in nursing homes*
Cluster 2	291	*W* ^0^ *C* ^0^	*Take, social, vaccinated, vaccine, paid, need*	*Only elderly should take the vaccine, elderly need social care, have paid their dues*
**Old folks**				
Cluster 1	325	*W* ^−^ *C* ^−^	*Die, take, shit, care, need*	*Old folks are going to die, don't have sufficient care insurance, need more benefits*
Cluster 2	269	*W* ^0^ *C* ^0^	*Early, use, home, life, time, lol*	*Old folks go to bed early, have plenty of time, enjoy life*
**Old people**				
Cluster 1	1,651	*W* ^−^ *C* ^−^	*Need, care, die, old, people, COVID*	*Old people die from COVID, are to die anyways, need social care, need to stay out of things*
Cluster 2	508	*W* ^−^ *C* ^−^	*Hate, annoying, rude, mad, fucking, shit*	*I hate old people doing this, old people hate skateboarders, are annoying, are rude and judgemental*
**Senior citizens**				
Cluster 1	376	*W* ^−^ *C* ^−^	*Stop, facing, risk, pay, money*	*Senior citizens are facing various problems, are at most risk for COVID, don't have enough money to pay utility and medical bills*
Cluster 2	406	*W* ^+^ *C* ^0^	*Day, senior, life, need*	*On the World Senior Citizens' day we honor our senior citizens, senior citizens need respectful life and happiness*

*The cluster locations on the warmth(W)–competence(C) plane are denoted as ^+^(pos), ^−^(neg), ^0^(neutral)*.

#### 4.3.2. Discussion

Our second case study confirmed two hypotheses from the related literature, namely: that the labels used to categorize different social groups are associated with different stereotypes, and that the COVID-19 pandemic has reinforced a view of older adults as frail and vulnerable. We discuss these two findings in more detail below.

**Connotative Meaning of Stereotype Labels**

An important aspect to group labeling, as described by Beukeboom and Burgers (2019), is what is considered acceptable according to prevailing social norms. Indeed, there is currently some debate over acceptable terms for referring to older adults as a group. Terms like *the elderly* may sound appropriate to some people, but many within the field of gerontology and elsewhere have argued that it constitutes “othering” language and should be avoided (Lundebjerg et al., 2017). Tellingly, very few people choose to refer to themselves as *elderly* (Berridge and Hooyman, 2020). Similarly, the term *senior citizen* has negative connotations for many people, although some may still use it with polite intentions.

However, *old people* is not generally considered a polite label, and so it is not surprising that users select this term when what they are saying about older people also violates social norms. The extreme low-warmth cluster, seen only with this label, seems to correspond most closely with the “shrew/curmudeon” stereotype characterized by Hummert et al. (1994) using words like *prejudiced, bitter, selfish*, and *nosy*.

**COVID-19 and the “Vulnerability” Stereotype**

As put succinctly by Berridge and Hooyman (2020), “The COVID-19 pandemic has highlighted the ease in which ageist language is employed and ageist stereotypes are used to characterize older adults.” While it is certainly true that many older adults suffered immensely due to the pandemic, researchers have pointed out the dangers to portraying older adults as a separate category of people who are “vulnerable” and “at-risk”: it encourages *us-vs.-them* thinking, it lumps together a large and highly heterogeneous group of people who face very different health-related risks, and it over-simplifies the cause of health outcomes to a single biological factor (age) instead of emphasizing social and economic inequalities, barriers to healthcare access, and problems of over-crowding and under-staffing in long-term care facilities.

### 4.4. Limitations

The two case studies present potential applications of the proposed technology to analyze stereotypical language “in-the-wild” in an unsupervised manner. While they demonstrate the benefits of the technology in practical settings, there are many limitations to this approach that should be acknowledged.

As mentioned above, using Twitter as a source of observational data rather than the survey-based approach of directly querying participants' known stereotypes leads to very noisy data. In an attempt to narrow down this stream of information to personal opinions about a target group, we applied several strategies for filtering and cleaning the texts; however, this process inevitably allowed some noise through while also removing some potentially relevant data. As well, we focus on texts where the target groups are mentioned as a whole, usually using plural nouns (e.g., *women, old people*). However, stereotypical thinking can be expressed when referring to the group using single nouns (e.g., “*A woman's place is in the kitchen.”*) or surface in discussions of particular group members, such as famous politicians, colleagues, neighbors, and so on.

Current NLP technologies, while showing significant improvements over the recent years, still have their limitations as well, especially when applied to noisy, real-life texts, such as social media. Twitter is notorious for the wide use of unconventional spelling and grammar, abbreviations, hashtags, and emojis. Language in general, and social media language specifically, is constantly evolving, as new terms or new meanings for existing terms emerge. This presents difficulties for pretrained language models and processing tools, which are usually trained on more conventional and structured types of texts. Also, recognizing and appropriately handling creative and figurative language, including sarcasm, humor, irony, and metaphors, has been a significant challenge for automatic processing techniques (Veale et al., 2016; Abulaish et al., 2020).

Finally, in this work we focus on processing only textual information. However, many tweets include images and short video clips that help users illustrate their points. Future work should incorporate processing of multi-modal inputs for a more comprehensive view of the content.

## 5. Conclusion

We have presented a computational approach to the Stereotype Content Model. In addition to validating the model on manually annotated data and specialized lexicons, we presented two case studies as demonstrations of how the method might be used to study and compare different stereotypes present in large text corpora, using a framework grounded in psychological theory.

Our approach uses pretrained embedding models and learns the direction of the SCM axes from a publicly available lexicon, annotated for warmth and competence. It is therefore computationally inexpensive and does not require extensive human annotations. Also, in contrast to word-level techniques, our model is applicable to many different types of sentences, and can handle semantic and syntactic complexities without additional pre-processing By expanding this computational model to a general framework for analyzing stereotypical language, we showed how this method can be used to process textual data about various target groups. In addition to presenting and discussing opinions frequently expressed by Twitter users about women and older adults, we contrasted our data mining approach with survey-based approaches and showed the discrepancies between what people describe as stereotypes when directly asked, with stereotypical views they spontaneously express on social media. Our results also demonstrate that stereotypes expressed by Twitter users might be different from those held by the society in general, due to the specific characteristics of this platform. Further, we show the significance of the labels people choose when referring to a group in conveying stereotypical views.

Identifying and analyzing stereotypes from real-life texts can help social scientists, non-profit organizations, and governments to track changes in society's views on various minority and historically marginalized groups, and intervene with educational and support campaigns and other preventive measures. On the other hand, such research might pose a risk of misuse or abuse by certain dominant groups to further marginalize and discriminate against minorities. Careful consideration of the potential impacts of such technology on various populations need to take place at every stage of the system design, development, and deployment. Still, we believe that the work on stereotype analysis can be highly beneficial for society.

In future work, we hope to explore how multiple ideas about a group (perhaps expressed across multiple sentences) combine to form complex and multi-faceted stereotypes, as expressed by individuals, groups of people, or on an institutional level. We are also interested in how stereotypes and their labels change over time, in response to changing social roles and cultural norms. As a long-term goal, we hope to better understand the motivations that lead to users posting stereotypical content online, and to develop methods of value-sensitive design to nudge them toward more inclusive and pro-social discourse.

## Data Availability Statement

The datasets generated and analyzed for this study can be found in public repositories. The seed lexicon created by Nicolas et al. (2021) is available at https://osf.io/yx45f/. The StereoSet dataset by Nadeem et al. (2020) is available at https://stereoset.mit.edu/. The test data used to generate Table 2 and the manual BWS annotations for warmth and competence described in Section 3.2.1 are available in the Supplementary Material. The code generated for this project is available at https://github.com/katiefraser/computational-SCM.

## Author Contributions

KF, SK, and IN contributed to the concept and design of the study. KF implemented the model and performed the validation. SK collected and pre-processed the Twitter data for the case studies. IN contributed to the analysis and interpretation of the results. All authors were active in the writing and revisions of the paper. All authors contributed to the article and approved the submitted version.

## Funding

This work was supported by the National Research Council Canada.

## Conflict of Interest

The authors declare that the research was conducted in the absence of any commercial or financial relationships that could be construed as a potential conflict of interest.

## Publisher's Note

All claims expressed in this article are solely those of the authors and do not necessarily represent those of their affiliated organizations, or those of the publisher, the editors and the reviewers. Any product that may be evaluated in this article, or claim that may be made by its manufacturer, is not guaranteed or endorsed by the publisher.

## Footnotes

^1^ https://github.com/katiefraser/computational-SCM

^2^We use the same notation as Mathew et al. (2020) to explain our method.

^3^https://huggingface.com/sentence-transformers (accessed July 14, 2021).

^4^“Use paraphrase-mpnet-base-v2 for the best quality”: https://www.sbert.net/docs/pretrained_models.html (accessed July 14, 2021).

^5^ https://stereoset.mit.edu/

^6^ https://spacy.io/
