# Supplementary material for: Computational Modeling of Stereotype Content in Text
Source: Front Artif Intell. 2022 Apr 19;5:826207. doi: 10.3389/frai.2022.826207 (PMC9063736; doi:10.3389/frai.2022.826207)
Supplement: Supplementary file 1 [file Data_Sheet_1.zip › Supplementary Material.PDF]

Supplementary Material

1 AXIS-ROTATED POLAR

Our motivation to develop axis-rotated POLAR arose from the observation of an apparent negative correlation between warmth and competence scores using the original POLAR framework. To visualize the differences between the standard POLAR and the axis-rotated POLAR models, we refer to Figure S1, which shows how test points for the 'basic functionality' and 'semantic composition' test cases are distributed across the plane for a single fold of the evaluation. Rotating the axes reduces the noticeable skew seen in Figures S1(a) and (c) and results in a more uniform, symmetrical distribution across the plane.

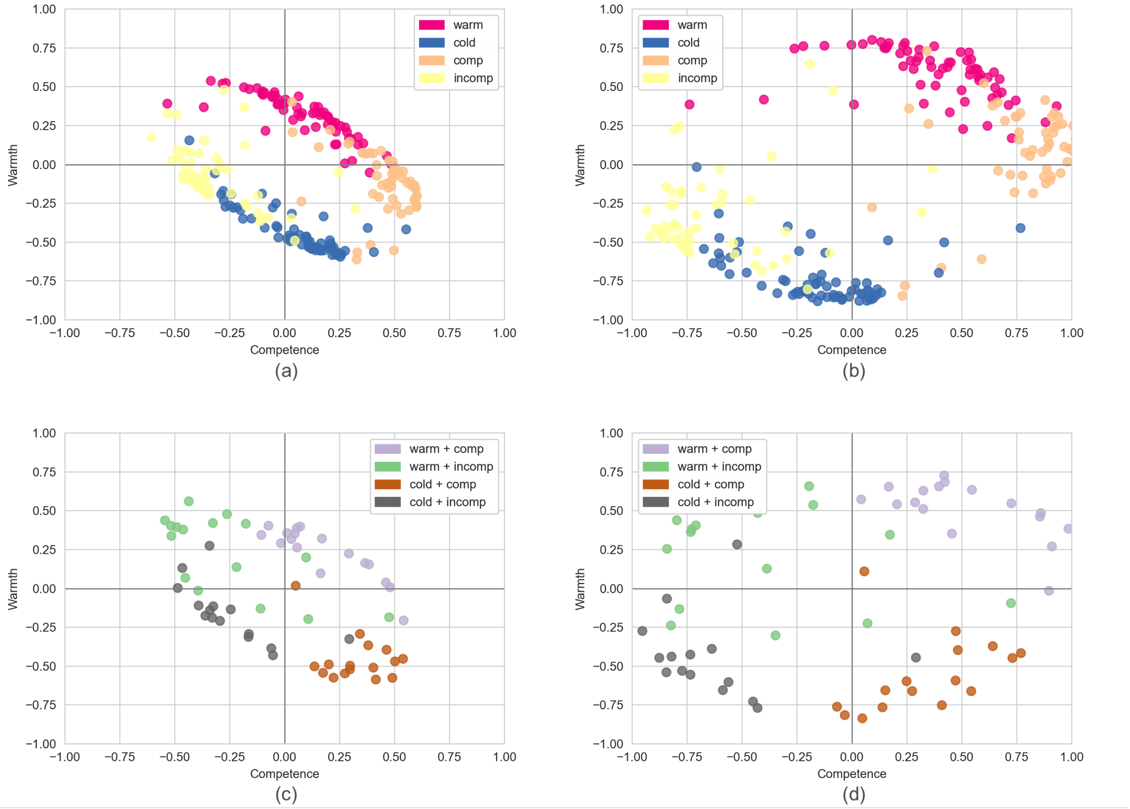

Figure S1. Comparing the distribution of test cases for the standard POLAR (left side) and rotated-axis POLAR (right side). Colour indicates the gold label of each test case. In subfigures (a) and (b), we show the locations of the test cases for basic functionality for a single fold. In subfigures (c) and (d), we show the locations of the test cases for semantic composition in a single fold.

2 BEST-WORST SCALING

Conventionally, to obtain human judgements on an ordinal scale, the annotators are asked to rate an item on a fixed 5- or 7-point scale (e.g., Likert scale). However, such annotations often suffer from inconsistencies in judgements among annotators as well as inconsistencies in responses of an individual annotator when annotating many items, since it can be hard to perceive differences between nearby points on the scale (Baumgartner and Steenkamp, 2001; Presser and Schuman, 1996). Further, some annotators tend to use

only part of a scale, for example avoiding the extreme points. To overcome these issues, comparative annotation schemes–where two or more items are compared with regard to the property of interest–can be used.

Best-Worst Scaling (BWS) is a comparative annotation method where an annotator is presented with n items, called an n-tuple (n is usually 4 or 5), and asked to select the *best* item and the *worst* item (e.g., a statement the annotator agrees with the most and a statement the annotator agrees with the least). All the items to be annotated are organized in n-tuples in such a way that ensures each item is annotated multiple times and compared with a diverse set of other items. After annotating around $2 \times N$ n-tuples, where N is the total number of items to be annotated, a real-valued score for each item and an overall ranking of items according to the property of interest can be obtained.

The three authors independently annotated all adjectives associated with sociability/morality (warmth) and ability/agency (competence) in the seed lexicon, in total 235 words. Each word was annotated for both warmth and competence, disregarding their original label in the seed lexicon. From the full set of words, 470 4-tuples were created so that each word appears in about 8 diverse tuples. For each 4-tuple, the thee annotators provided answers to four questions:

1. Which word is associated with being friendly/social the most? (HIGHEST WARMTH)
2. Which word is associated with being friendly/social the least? (LOWEST WARMTH)
3. Which word is associated with being competent/able the most? (HIGHEST COMPETENCE)
4. Which word is associated with being competent/able the least? (LOWEST COMPETENCE)

Note that the same word could be chosen as HIGHEST/LOWEST WARMTH and HIGHEST/LOWEST COMPETENCE, but no word could be selected as both HIGHEST and LOWEST WARMTH, or both HIGHEST and LOWEST COMPETENCE. If two or more words were judged as equally associated with a dimension, then annotators were instructed to choose between them randomly, resulting in similar scores for such words.

Then, a score for each item for the warmth dimension was calculated as the difference between the proportion of times the item has been selected for HIGHEST WARMTH and the proportion of times the item has been selected for LOWEST WARMTH (Flynn and Marley, 2014; Kiritchenko and Mohammad, 2016). The scores for the competence dimension were calculated in a similar manner using HIGHEST and LOWEST COMPETENCE answers.

We assess inter-annotator agreement using Spearman's rank correlation since we are most interested in the relative rankings of words along each axis. The correlation between each pair of the annotators was high, with warmth scores being slightly more highly correlated (mean $\rho = 0.886$) than competence scores (mean $\rho = 0.851$). This may be related to previous findings that warmth is the primary dimension of social cognition, is more heavily weighted in forming impressions, and is more cognitively accessible than competence (Fiske et al., 2006; Wojciszke et al., 1998).

Examining the words on which there was low agreement amongst the annotators was also informative. For example, the word *discriminating* proved to be ambiguous, with some annotators interpreting it in the sense of *one having refined taste* and others interpreting it as *one showing prejudice against a social group*. Other words differed in the perceived degree of competence (e.g., *shrewd, vulnerable, dishonest, shy*) or warmth (e.g. *popular, assertive, wise, cunning*). Furthermore, the words were presented in a neutral context, and may take on different shades of meaning in different sentences. However, given the generally high agreement amongst annotators, we consider the scores sufficient for this validation study.

3 TWITTER DATA COLLECTION

The collection was conducted for one month, from August 20, 2021 until September 20, 2021. Since our goal is to gather personal opinions of individual users, we discard re-tweets, tweets containing URLs to external websites, and tweets with five or more hashtags. We further attempt to automatically identify accounts belonging to bots and organizations, and discard all tweets posted by those and other prolific accounts. We automatically identify bots and organizations as Twitter accounts containing the word *bot* or target group related words in their user name or screen name. For organizations associated with women, we use the words *women* and *female*, and for organizations associated with older adults, we use the words *elderly* and *senior*. Other prolific accounts are identified as accounts with more than N tweets in our one-month collection. After manual examination of the accounts with the most posts, we set N to 600 for tweets mentioning women query terms and 150 for tweets collected for the age-related groups.

4 HDBSCAN PARAMETERS

There are a number of free parameters in the HDBSCAN algorithm, which we set as follows: the *min samples* parameter (the number of samples in a neighbourhood for a point to be considered a core point) is set to twice the dimensionality of the data, i.e., four, following the heuristic laid out by Schubert et al. (2017). From the same paper, we follow the procedure to determine the distance threshold parameter ϵ from the k-nearest neighbours plot. Finally, there is an optional parameter to control the minimum cluster size. As we are interested here in discovering the most common and highly-frequent perceptions of various groups, we select a relatively high minimum cluster size, requiring each cluster to contain at least 1–2% of the total data for a given target group. In other applications, this parameter could be set lower (resulting in a greater number of smaller clusters) or higher (resulting in a fewer, larger clusters).

5 POINTWISE MUTUAL INFORMATION

For cluster interpretation, we calculate an association score of a word w with the target cluster C_{target} as compared to the rest of the corpus $C_{reference}$ (i.e., all the other clusters for the group combined), using Pointwise Mutual Information (PMI):

$$Score\,(w) = PMI\,(w, C_{target}) - PMI\,(w, C_{reference}) = log_2 \frac{freq\,(w, C_{target}) * freq\,(C_{reference})}{freq\,(w, C_{reference}) * freq\,(C_{target})} \tag{S1}$$

where $freq\,(w, C_{target})$ and $freq\,(w, C_{reference})$ are the numbers of times the word w occurs in the target cluster and in all the other clusters, respectively, and $freq\,(C_{target})$ and $freq\,(C_{reference})$ are the total numbers of words in the target cluster and in all the other clusters.

Words with $Score\,(\mathrm{w}) \geq 0.6$ (i.e., words whose frequency in the target cluster is at least $1.5 \times \frac{freq\,(C_{target})}{freq\,(C_{reference})}$ times higher than in the reference corpus) represent the corresponding cluster. We ignore stopwords and low-frequency words. The frequency threshold is set up to 3% of the number of sentences in the corresponding target corpus, but not smaller than 3.

6 DISTRIBUTION OF WARMTH AND COMPETENCE SCORES

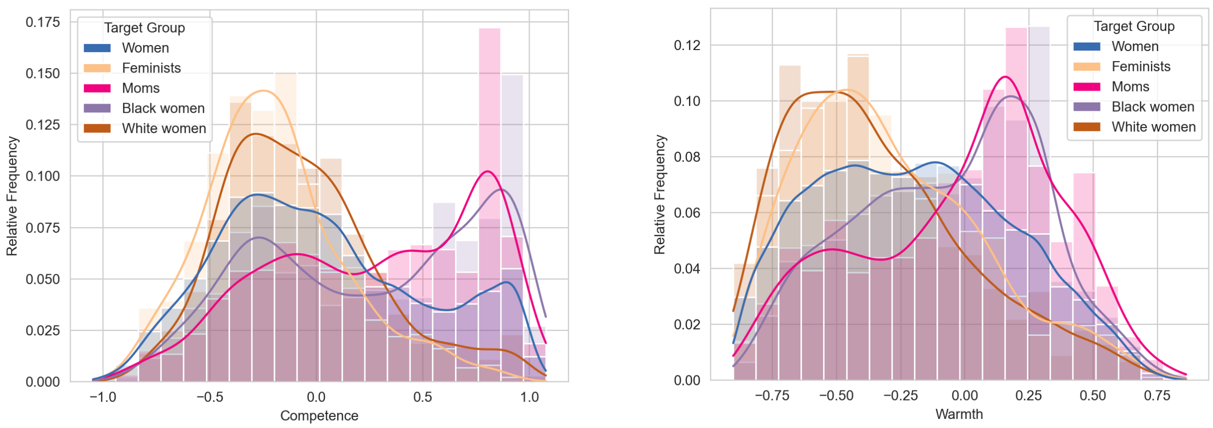

Figure S2. Distributions of competence and warmth values for different sub-groups of women.

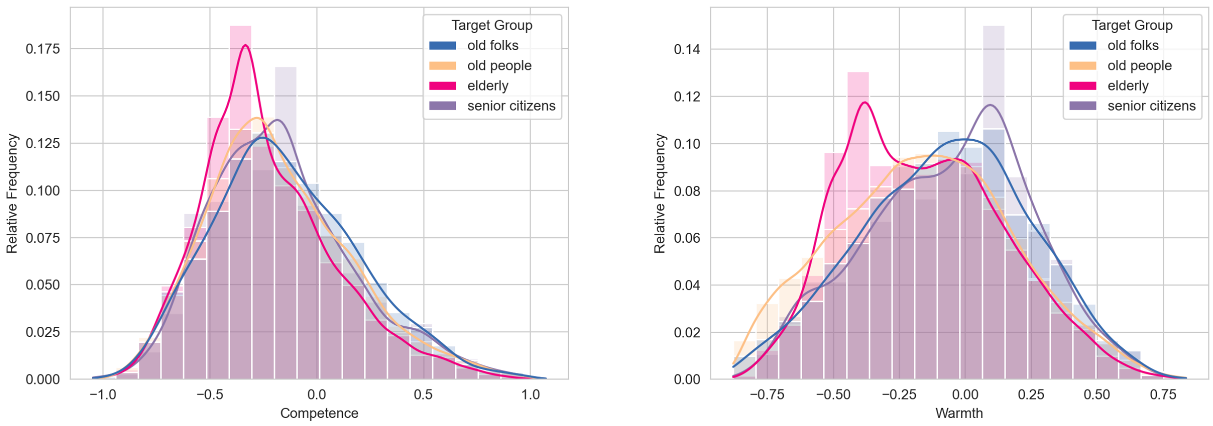

Figure S3. Distributions of competence and warmth values for different labels for older adults.

REFERENCES

Baumgartner, H. and Steenkamp, J.-B. E. (2001). Response styles in marketing research: A cross-national investigation. *Journal of Marketing Research* 38, 143–156

Fiske, S. T., Cuddy, A. J. C., and Glick, P. (2006). Universal dimensions of social cognition: Warmth and competence. *Trends in Cognitive Sciences* 11, 77–83

Flynn, T. N. and Marley, A. A. J. (2014). Best-worst scaling: theory and methods. In *Handbook of Choice Modelling*, eds. S. Hess and A. Daly (Edward Elgar Publishing). 178–201

Kiritchenko, S. and Mohammad, S. M. (2016). Capturing reliable fine-grained sentiment associations by crowdsourcing and best–worst scaling. In *Proceedings of the Annual Conference of the North American Chapter of the Association for Computational Linguistics: Human Language Technologies (NAACL)* (San Diego, California)

Presser, S. and Schuman, H. (1996). *Questions and Answers in Attitude Surveys: Experiments on Question Form, Wording, and Context* (SAGE Publications, Inc)

Schubert, E., Sander, J., Ester, M., Kriegel, H. P., and Xu, X. (2017). DBSCAN revisited, revisited: Why and how you should (still) use DBSCAN. *ACM Transactions on Database Systems (TODS)* 42, 1–21

Wojciszke, B., Bazinska, R., and Jaworski, M. (1998). On the dominance of moral categories in impression formation. *Personality and Social Psychology Bulletin* 24, 1251–1263
